# Continuum models of focused electron beam induced processing

**DOI:** 10.3762/bjnano.6.157

**Published:** 2015-07-14

**Authors:** Milos Toth, Charlene Lobo, Vinzenz Friedli, Aleksandra Szkudlarek, Ivo Utke

**Affiliations:** 1 School of Physics and Advanced Materials, University of Technology, Sydney, 15 Broadway, Ultimo, New South Wales 2007, Australia; 2 Empa, Swiss Federal Laboratories for Materials Science and Technology, Laboratory for Mechanics of Materials and Nanostructures, Feuerwerkerstrasse 39, 3602 Thun, Switzerland; 3 AGH University of Science and Technology, Academic Centre for Materials and Nanotechnology, al. A. Mickiewicza 30, 30-059 Krakow, Poland

**Keywords:** continuum model, deposition, electron beam processing, etching, gas injection system

## Abstract

Focused electron beam induced processing (FEBIP) is a suite of direct-write, high resolution techniques that enable fabrication and editing of nanostructured materials inside scanning electron microscopes and other focused electron beam (FEB) systems. Here we detail continuum techniques that are used to model FEBIP, and release software that can be used to simulate a wide range of processes reported in the FEBIP literature. These include: (i) etching and deposition performed using precursors that interact with a surface through physisorption and activated chemisorption, (ii) gas mixtures used to perform simultaneous focused electron beam induced etching and deposition (FEBIE and FEBID), and (iii) etch processes that proceed through multiple reaction pathways and generate a number of reaction products at the substrate surface. We also review and release software for Monte Carlo modeling of the precursor gas flux which is needed as an input parameter for continuum FEBIP models.

## Review

### Introduction to continuum models of focused electron beam induced processing (FEBIP)

Continuum FEBIP models enable the simulation of process rates that govern focused electron beam induced etching (FEBIE), deposition (FEBID) [1–16] and surface functionalization [17] techniques. They are typically used to simulate growth rates and nanostructure geometries as a function of experimental parameters, and to help elucidate the underlying growth mechanisms. Continuum FEBIP models are comprised of differential equations for the rates of change of concentrations of all surface-adsorbed species thought to be involved in the deposition or etch kinetics. The rate equations are functions of time and space, and require specification of the molecular properties of each adsorbate and the electron flux profile(s) at the solid–vacuum interface. Simple continuum FEBIP models can be solved analytically, yielding governing laws delineating the so-called “reaction-rate” and “mass transport” limited process regimes, and resolution scaling laws. Numerical models can account for adsorbate diffusion and enable modeling of processes such as simultaneous FEBIE and FEBID performed using a mixture of precursor gases. Here we provide software that can be used to simulate a wide range of processes reported in the FEBIP literature, and review the underlying continuum FEBIP models (recent general reviews of FEBIP can be found in [4,10,18–21]). We begin with a discussion of the reaction rate limited regime and the most common continuum model input parameters: initial adsorbate coverage, electron flux profile and the gas flux distribution produced by a capillary-style gas injection system. We then cover simple continuum models that are valid in the reaction rate limited regime (where net adsorbate transport via surface diffusion is negligible) and can be used to model FEBIP performed using continuous and pulsed electron beams, physisorbed and chemisorbed precursor molecules, gas mixtures, and multiple reaction products. Finally, we cover a number of models that account for surface diffusion and can be used to model FEBIP in both the reaction and mass transport limited regimes. Throughout, we emphasize the underlying assumptions and limitations inherent to each model.

Before beginning our discussion, we note that the terms “rate”, “concentration” and “flux” are always used to describe quantities with units of reciprocal time [s^−1^], reciprocal area [Å^−2^] and their product [Å^−2^s^−1^], respectively. For example, the concentration of adsorbate species ‘a’, *N*_a_, their desorption rate *k*_a_ and desorption flux *N*_a_*k*_a_ have units of [adsorbates/Å^2^], [molecules/s] and [molecules/Å^2^/s], respectively. Frequently used symbols in this review are defined in Table 1. The term “growth” is applied to both FEBIE and FEBID (positive and negative growth rates refer to the growth of deposits and etch pits, respectively). We limit our discussion to FEBIP performed using a stationary, continuous or pulsed, radially symmetric electron beam (i.e., models implemented in cylindrical coordinates). Examples of models of scanned beams can be found in [22–24].

**Table 1:** Guide to commonly used symbols.

symbol	units	definition

Θ	Å^−2^	adsorbate coverage
Λ	Å^−2^s^−1^	adsorption flux
Ω	Å or nm	standard deviation of a Gaussian beam
δ	Å	molecule diameter
κ	s^−1^	desorption attempt frequency
κ_D_	Å^−2^s^−1^	diffusion coefficient pre-factor
λ	m	mean free path
σ	Å^2^	cross-section
τ	s^−1^	residence time
*A*	Å^2^	adsorbate area
*D*	Å^2^s^−1^	diffusion coefficient
*E*	eV	energy
*F* or *J*	Å^−2^s^−1^	gas molecular flux
*N*	Å^−2^	adsorbate concentration
*P*	Pa or mbar	gas pressure
*T*	K	substrate temperature
*T*_g_	K	gas temperature
*V*	Å^3^ or nm^3^	volume
*d*	μm	GIS capillary diameter
*f*	Å^−2^s^−1^	electron flux
*h*	Å or nm	height
*k*	s^−1^	desorption rate
*k*_B_	eV·K^−1^	Boltzmann’s constant
*n*_0_	Å^−2^	maximum (monolayer) adsorbate concentration
*s*	N/A	sticking probability

#### Reaction rate limited growth regime

In order to reach clear conclusions it is often desirable to perform simulations and experiments under simplified conditions where one or more processes are negligible. In the case of FEBIP models, a useful simplification occurs when the net transport of adsorbates via surface diffusion is negligible. This condition can be met by performing FEBIP in the so-called reaction rate limited growth regime (also called the electron-limited growth regime) where the extent of adsorbate depletion caused by electrons is negligible [4,10,19–21]. The strict definition of reaction rate limited growth is that the electron-induced dissociation rate is much smaller than the sum of the adsorption rate and the thermal desorption rate, i.e., using the symbols defined below and in Table 1, for adsorbate species ‘a’, 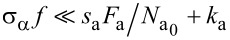. Conversely, the mass transport limited growth regime (also called the adsorbate-limited growth regime) is defined as 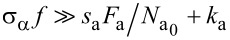. In the mass transport limited regime, the magnitude of the electron flux is sufficiently high to cause significant depletion of precursor adsorbates, and adsorbate diffusion into the area irradiated by electrons makes an important contribution to the growth rate.

**Figure 1:**
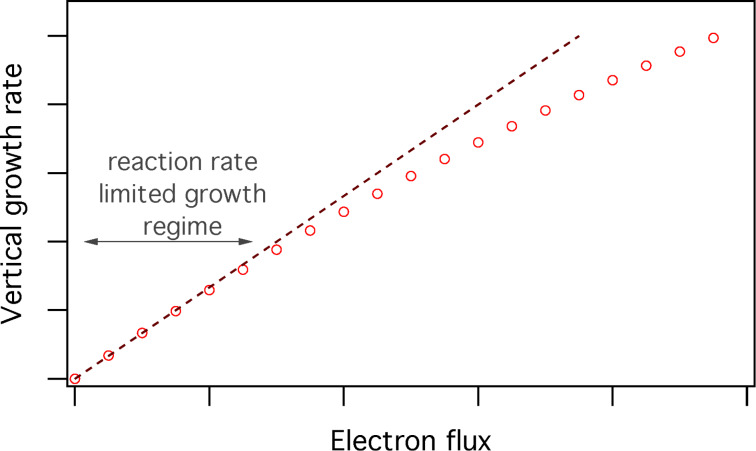
Steady state vertical growth rate of a deposit plotted as a function of electron flux. The linear region of the curve in the limit of low electron flux corresponds to the reaction rate limited growth regime.

In practice, the reaction rate limited regime can be identified simply by measuring or simulating the steady state growth rate as a function of electron flux, as illustrated in Figure 1 (in the figures, numerical values are excluded from axis labels when the plots are used to illustrate general trends that occur under a wide range of FEBIP conditions). Here, the linear portion of the curve corresponds to the reaction rate limited growth regime. In the mass transport limited growth regime, the deposit/pit shapes can provide information on the role of diffusion in the FEBIP process. Inclusion of diffusion in continuum FEBIP models adds a layer of complexity to the modelling and to interpretation of the model outputs. It is necessary only if diffusion is a major contribution to the growth rate. We will discuss models that incorporate diffusion in a self-contained section below, but will limit the majority of our discussion to diffusion-less models.

#### Initial adsorbate coverage

**Figure 2:**
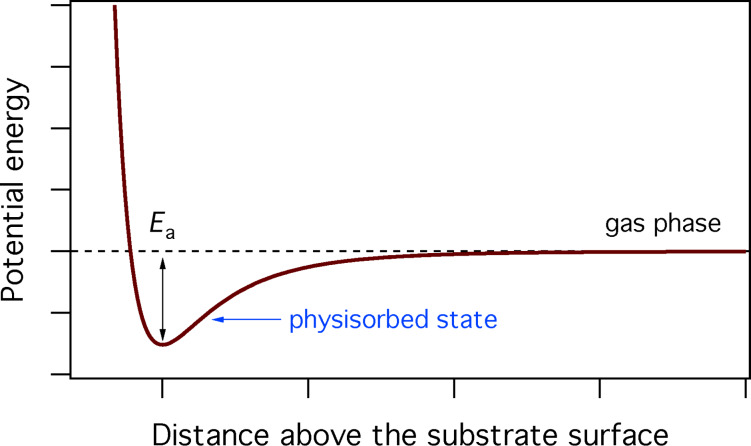
Potential energy diagram for adsorption governed by a single potential well at the surface. Modified from [18].

FEBIP models require specification of the initial concentration of surface-adsorbed precursor molecules 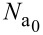, i.e., the steady-state concentration of adsorbates in the absence of electron irradiation. 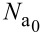 can be found by solving for the difference between the flux of molecules adsorbing from and returning to the gas phase. In the gas phase, the molecule flux is given by:

[1]
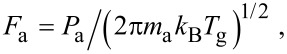


where *P*_a_ is the pressure of the precursor gas for adsorbate ‘a’, *m*_a_ is the mass of a gas molecule, *k*_B_ is Boltzmann’s constant and *T*_g_ is the gas temperature. The simplest case of gas-molecule adsorption onto a substrate surface is that of physisorption, described by a single potential well at the surface as shown in Figure 2. The flux Λ_a_ of precursor molecules physisorbing to vacant surface sites is given by:

[2]
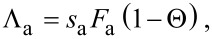


where *s*_a_ is the sticking coefficient (in the limit of zero surface coverage), and Θ is the adsorbate coverage, i.e., the fraction of surface sites occupied by physisorbed gas molecules:

[3]
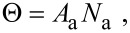


where *A*_a_ is the area of a surface site occupied by adsorbate ‘a’, and 1/*A*_a_ is the maximum possible concentration of species ‘a’.

We note that Equation 2 describes non-activated Langmuir adsorption of a single molecular species ‘a’. The Langmuir model limits the surface coverage to one monolayer (hence the term (1 − Θ) in Equation 2), and can be modified to account for other adsorption behavior such as multilayer adsorption and thermally activated chemisorption. We also note that most FEBIP models assume that *s*_a_ is independent of temperature.

The thermal desorption rate *k*_a_ of the physisorbed species ‘a’ is given by:

[4]
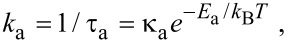


where τ_a_ is the adsorption time (i.e., adsorbate residence time), κ_a_ is the desorption attempt frequency, *E*_a_ the desorption energy, (i.e., the depth of the potential well shown in Figure 2), and *T* is the temperature of the substrate surface. The thermal desorption flux is given by *N*_a_*k*_a_, and the adsorbate concentration is found by solving

[5]
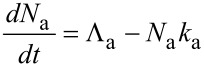


for *N*_a_(*t*):

[6]
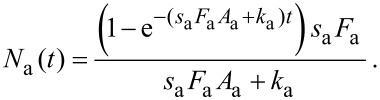


**Figure 3:**
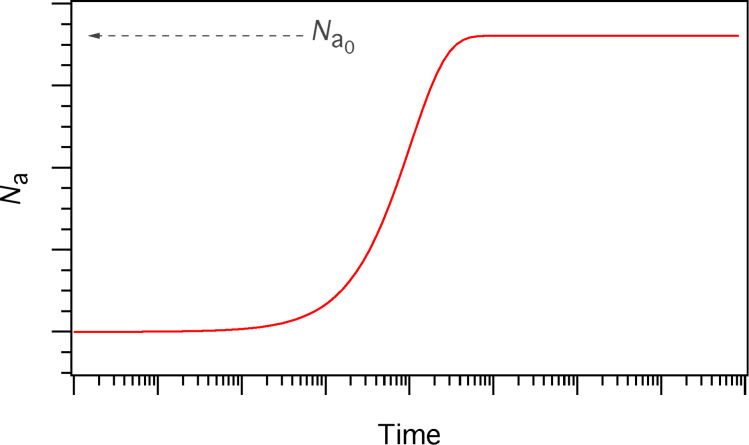
Adsorbate concentration (*N*_a_) versus time in the absence of electron irradiation (here, *N*_a_ = 0 at *t* = 0). 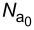 is the steady state adsorbate concentration in the absence of electron irradiation, which is used as the initial adsorbate concentration input into FEBIP models. *N*_a_(*t*) and 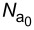 are given by Equation 6 and Equation 7, respectively.

In Equation 6, *t* = 0 represents the time at which the gas flux *F*_a_ is turned on in the model, i.e., the time at which the gas pressure is changed from 0 to *P*_a_. A typical time-evolution of *N*_a_ in the absence of electron irradiation is shown in Figure 3. As *t* → ∞, the surface coverage reaches a steady-state equilibrium value, 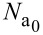, which is the initial value that is input into FEBIP models (in which *t* = 0 represents the time at which the electron flux is turned on):

[7]
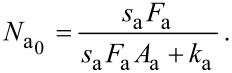


#### Electron flux profile

Spatially-resolved FEBIP models require specification of the electron flux profile *f*(*r*). Focused electron beams are usually approximated by a Gaussian function:

[8]
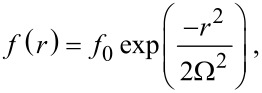


where Ω is the standard deviation (i.e., full width at half maximum, FWHM = 
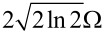
) of the Gaussian beam. Defocused beams typically have a tophat shape that can be approximated by:

[9]
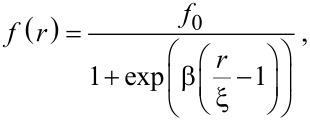


where *f*_0_ is the (maximum) flux at the beam axis (*r* = 0), β defines the abruptness of the edge of the tophat, and ξ is the beam radius. Tophat beams are useful for quantitative analyses and modeling of experimental data because they are easy to measure and control with a high degree of accuracy [2–3,6,25–26].

**Figure 4:**
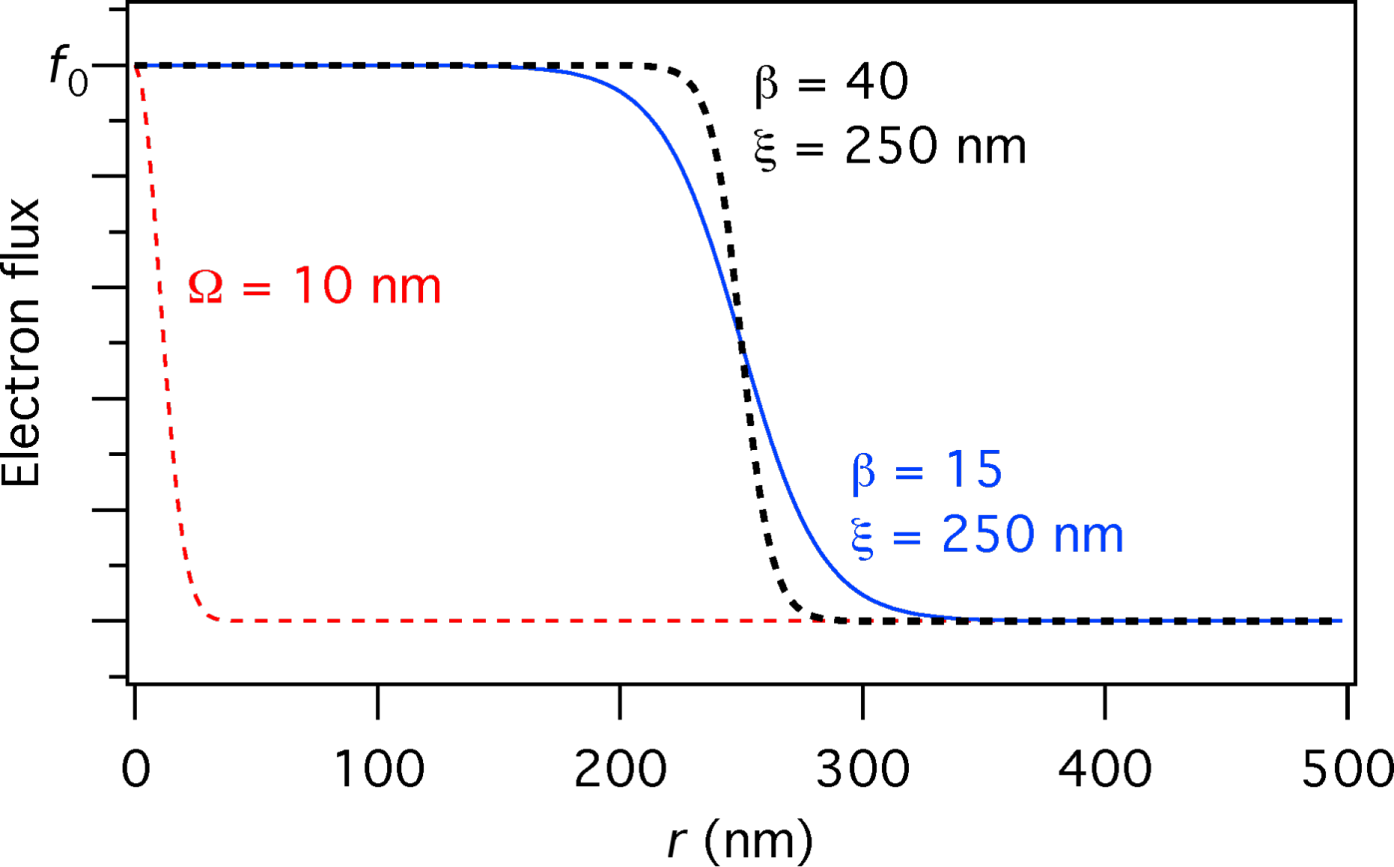
Gaussian electron flux profile (Ω = 10 nm) and two tophat flux profiles with a radius of 250 nm (β = 15 and 40). All three profiles are normalized to *f*_0_, the electron flux at the beam axis.

A Gaussian and two tophat electron flux profiles are shown in Figure 4. These profiles can be used to approximate those encountered in FEBIP. Actual flux profiles have contributions from primary, backscattered and secondary electrons, each of which has a unique spatial profile and a unique energy distribution [19].

### Gas flow from a capillary-style gas injection system (GIS)

FEBIP precursor gases are injected into a specimen chamber using one of two methods. In the first method, the entire vacuum chamber, or a sub-chamber [6] is filled with a precursor gas, as is done in environmental electron microscopy [27–30]. Such vacuum systems can be configured so that the gas pressure is uniform across the substrate surface, and can be measured accurately by conventional pressure gauges. However, in the vast majority of FEBIP setups, a gas injection capillary is used to inject the precursor gas into a chamber that is pumped continuously by a high-vacuum pumping system. The low conduction of the capillary makes it the element that limits the flow rate and shapes the flux profile of such gas injection systems. The capillaries are useful because they simultaneously assure a high local molecule flux at the substrate surface, and a low vacuum chamber background pressure, as required for robust operation of the vacuum system. However, a disadvantage of a capillary-style GIS is that the gas pressure varies dramatically throughout the chamber, and the gas pressure at the substrate surface cannot be measured accurately using conventional pressure gauges. Hence, in this section, we describe a Monte-Carlo simulator developed at Empa (the “GIS simulator”) for calculating gas pressure distributions generated by a capillary-style GIS. The code can be used to calculate the gas molecule flux at the beam impact point on the substrate surface (which is an input parameter to all FEBIP models).

Gas molecule impingement rates on the substrate have lateral distributions that depend on the capillary nozzle geometry, the angle and distance between capillary and substrate, and the molecule flow regime. The GIS simulator code enables the selection of some pre-defined nozzle geometries, capillary inner and outer diameters, angles and distances to the substrate, and either molecular or transient flow regime. By default it maps the impingement distribution on planar substrates, the molecule flux along the tube, and the angular nozzle effusion distribution. The code was validated by capillary injection experiments for both flow regimes [31–32].

Due to the Monte Carlo code implementation, the GIS simulator does not account for gas pressure gradients inside the capillary and above the substrate. The code can be used in an executable version or an editable version (http://www.empa.ch/febipcode) to include specific shadow effects by non-planar substrate geometries (such as deposits grown by FEBID), surface gas phase collisions, or other capillary geometries.

#### Flow regimes

To make a correct choice of the flow regime for the simulation one needs to determine the mean free path λ between molecule–molecule collisions and the Knudsen number (the ratio of λ to the capillary diameter *d*):

[10]
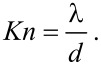


The Knudsen number specifies the ratio of wall collisions to molecule–molecule collisions. A value of 10 < *Kn* < ∞ signifies rare flow conditions under which the molecule gas flow distribution is determined predominantly by tube wall collisions (molecular flow regime). In the range 0.1 < *Kn* < 10 collisions between molecules become more important for shaping the flow (transient flow regime). When *Kn* < 0.1 the flow becomes viscous and can be treated by continuum models; this regime is not covered by the GIS simulator.

The calculation of the mean free path along a capillary is not trivial as the pressure *P* along the capillary is not constant. There will be a pressure gradient along capillaries directing net flow from the precursor reservoir (*P*≈ vapour pressure) to the vacuum chamber (*P*≤ 10^−4^ mbar) [33]. The commonly known relation 
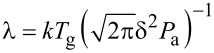
, where δ is the molecule diameter and other variables are as defined in Equation 1, can only be used if the pressure close to the capillary exit is known.

Mean free paths for a given GIS can be obtained by monitoring the precursor consumption rate *Q* (molecules per unit time) experimentally and calculating the mean free path using

**Figure 5:**
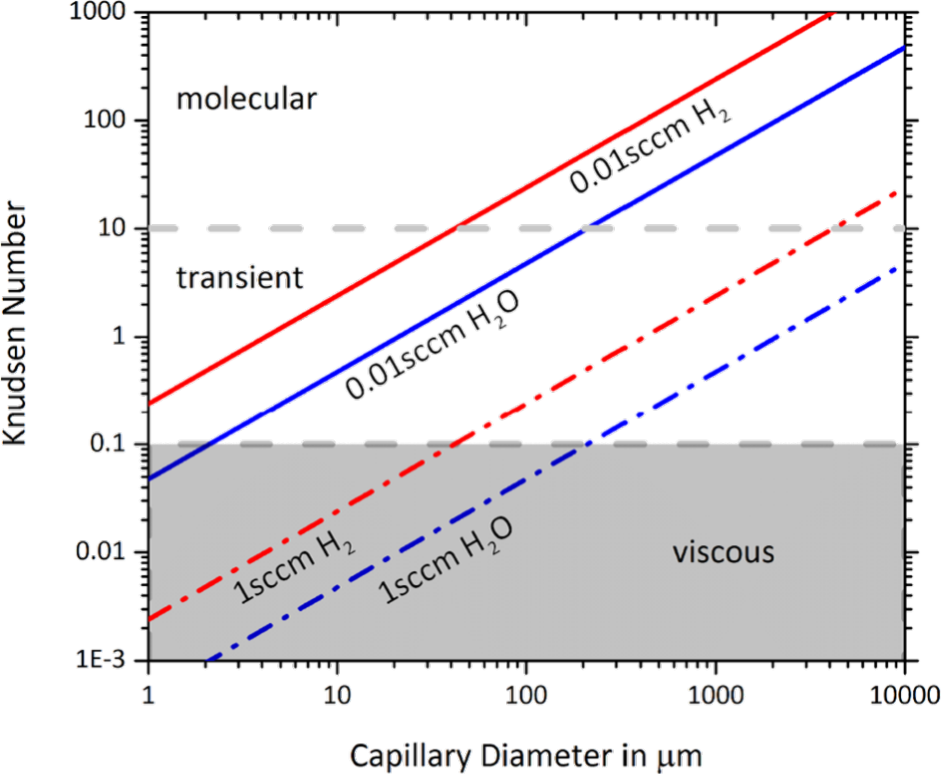
Molecule flow regimes for two flow rates *Q* of H_2_ and H_2_O. Note that 1 sccm = 4.48 × 10^17^ molecules/second, using Avogadro’s number and the standard volume of 22.4 L/mol of an ideal gas at 0 °C and 1 atm.

[11]
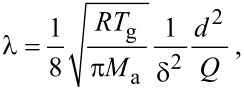


where *R* is the ideal gas constant and *M* is the molar mass [33]. The Knudsen number can then be calculated by inserting Equation 11 into Equation 10. The consumption rate of the precursor can be monitored by measuring its mass or volume change due to evaporation during FEBIP. Alternatively, mass flow controllers can be used to supply a defined flow rate *Q* (sccm). Figure 5 shows the dependence of the Knudsen number on the pipe diameter for two flow rates of H_2_ and H_2_O. At a fixed flow rate and capillary diameter, the Knudsen numbers and mean free paths scale with 
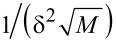
. Values for a few molecules are given in Table 2 together with their vapour pressure and monolayer adsorbate concentration *n*_0_ = 1/*A*_a_ = 1.154δ^−2^.

**Table 2:** Summary of molecule diameters (δ), molar masses (*M*), vapour pressures *P*_vap_ and monolayer adsorbate concentrations *n*_0_ of selected FEBIP precursors. The latter were calculated using *n*_0_ = 1.154δ^−2^.

precursor	*M* [g/mol]	δ [Å]	*n*_0_ [nm^−2^]	*P*_vap_ [Pa/*T*]	*P*_vap_ ref.

Me_3_PtCpMe	319.17	7.8^a^	1.9	19/31 °C	[34]
TEOS:Si(OEt)_4_	208.33	8.1^b^	1.8	172/25 °C	[36]
XeF_2_	169.29	4.5^b^	5.7	598/25 °C	[37]
Me_2_Au(tfac)	310.03	3.5^a^	9.4	7.3/23 °C	[38]
W(CO)_6_	351.90	6.8^b^	2.5	3.5/25 °C	[39]
Co_2_(CO)_8_	341.95	7^a^	2.4	0.6...16/20 °C^c^	[40]
Cu(hfac)_2_	477.65	8.0^b^	1.8	0.4/25 °C^d^	[41]
(hfac)Cu(VTMS)	370.83	8.6^b^	1.6	10/23 °C	[42]
[(PF_3_)_2_RhCl]_2_	628.48	5.7^a^	3.6	7.5/23 °C	[38]
O_2_	16	3.7^e^	8.4	—	—
H_2_	2	2.7^c^	15.8	—	—
H_2_O	18	3.5^b^	9.4	2330/20 °C	[44]

^a^Longest dimension of molecule. ^b^The molecule size is determined from the compound density ρ according to δ = 1.122(*M*/(ρ*N*_A_))^1/3^ where *N*_A_ is Avogadro’s constant [35]. ^c^Co_2_(CO)_8_ can disproportionate spontaneously in vacuum [40]. ^d^Cu(hfac)_2_ exists as dihydrate Cu(hfac)_2_·2H_2_O if not dried in vacuum with little effect on the vapour pressure [41]. ^e^Apparent diameters from [43].

To estimate an upper limit for the impingement rate, consider a substrate placed directly in front of a capillary with an exit area of 1 mm^2^ and a flow rate of 1 sccm. This results in a molecule flux *F*_a_ ≈ 4.5 × 10^19^ molecules cm^−2^s^−1^ leaving the capillary, or *F*_a_ ≈ 5 × 10^5^ monolayers per second (taking 10^14^ cm^−2^ as monolayer coverage). At a minimum, impingement rates of FEBIP molecules should be greater than the impingement rates of residual gases (at least one monolayer per second at a background pressure of 10^−6^ mbar). Of course, the molecule flux will rapidly decrease with distance between the capillary and the substrate.

#### Wall uptake

The GIS simulator incorporates an uptake factor for capillary wall collisions. Setting this value to zero means that all molecules colliding with the wall will immediately desorb; setting the value to 1 means that all molecules colliding with the wall will adsorb permanently (i.e., only molecules without wall collisions will exit the capillary). In most cases the uptake factor can be set to zero, as molecule condensation on capillary walls is normally avoided through the pressure gradient and keeping the reservoir at a temperature that is lower than or equal to that of the capillary walls. However, some organometallic molecules can spontaneously decompose in contact with the wall material and thus provide a surface for continuous autocatalytic decomposition through successive molecule–wall collisions. (Metal carbonyls are known for autocatalytic decomposition but data quantifying the uptake coefficients is sparse. A change in colour of the capillary points to such a mechanism). Analogous to the atomic layer deposition process [45], a more likely scenario is that at room temperature the molecules decompose partially via chemisorption upon collisions with unoccupied adsorption sites on the wall. This process is self-limiting after a short transient time during which all wall sites are occupied and the wall is passivated for further molecule decomposition.

#### Nozzle geometries

**Figure 6:**
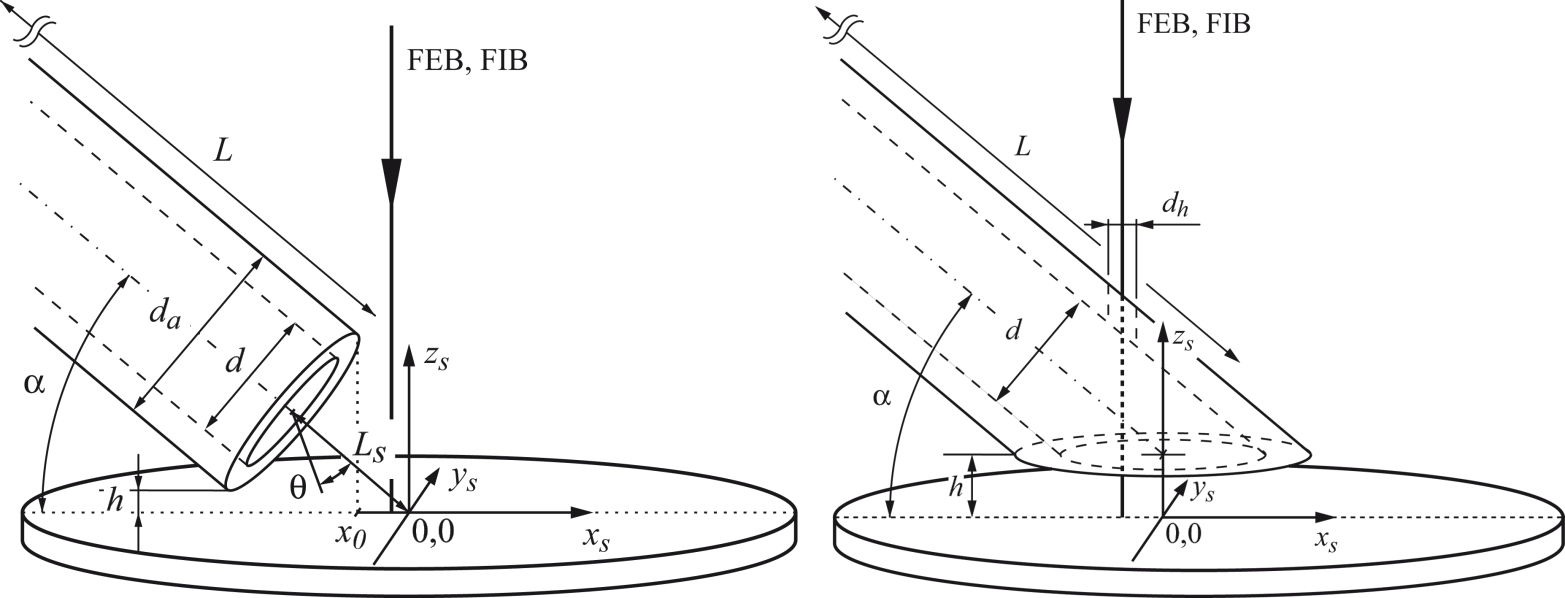
Illustration of the two capillary nozzle geometries implemented in the GIS simulator. Left: straight capillary; right: bevelled capillary. Modified from [31].

Two nozzle geometries are incorporated in the GIS simulator [31], and users can implement other geometries. Here we release the source code of version 1.5 containing the geometries shown in Figure 6 for straight capillaries and for bevelled capillaries with access holes. This C++ code is the base of the executable GIS simulator tool downloadable at http://www.empa.ch/febipcode. We would like to note that the code is not professionally commented nor written but has been checked against analytical solutions (for simple geometries) and against experiments. The current version of the code does not consider desorption of molecules from the substrate.

#### Simulation method

The physics behind the GIS simulator is described in detail in [31,33,46] and we give only a brief summary here. The code is based on the so called test-particle Monte Carlo method, which works in the molecular and transient flow regimes. The molecule flux distribution is obtained as a function of the nozzle geometry and the arrangement of nozzle and substrate. Molecule trajectories are computed consecutively for 10^6^ to 10^7^ molecules. This approach is strictly only valid for molecular flow conditions where molecule trajectories are independent of each other, and collisions occur only with the inner tube wall. However, it was shown experimentally [31] and by comparison with direct simulation Monte Carlo [33] that the test-particle Monte Carlo method with intermolecular collisions gives excellent results in the following way (points 3 and 4 are simplifications, which can be changed by the user if required):

1. Transient flow simulations can be performed by setting the mean free path λ = *d*·*Kn* inside the entire capillary. The mean free paths should be calculated from precursor consumption measurements using Equation 11. The flow regime prevailing near the end of the capillary exit will then be entered correctly into the simulation.
2. Wall collisions are implemented such that the molecules leave the wall with a cosine distribution in space, while molecule–molecule collisions are implemented as a uniform angular distribution. The capillary entry distribution is implemented as a cosine point source. The implementation of these angular distributions of wall and molecule collisions was shown to be correct by independent experiments [31–32].
3. Outside of the nozzle the Knudsen number is set to infinity, i.e., molecules follow straight trajectories from the last collision inside the capillary until they hit the substrate (i.e., there are no molecule–molecule collisions outside the tube).
4. Consecutive trajectories inside the vacuum chamber are not taken into account (molecule desorption is neglected).

The capillary length must be entered into the simulation. For long capillaries this is an enormous computational effort. A reduced capillary length *L*_r_ can be entered instead, still giving accurate results. For molecular flow conditions, the spatial distribution of impinging molecules on the substrate is not significantly altered for lengths *L*_r_ > 15*d*. For transient flow conditions, the reduced length depends on λ with *L*_r_ = 3λ to 5λ [31,33].

#### Simulation examples with the GIS simulator

**Figure 7:**
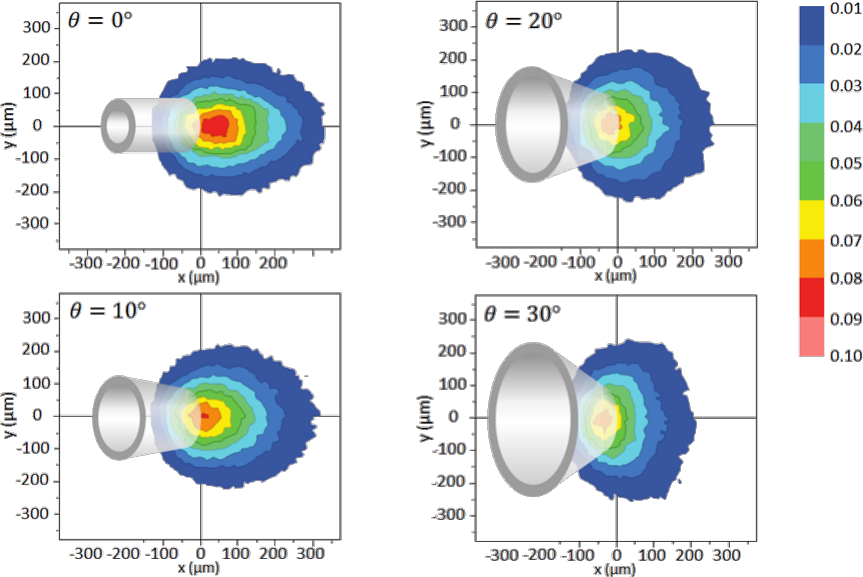
Precursor flux distributions at the substrate under molecular flow conditions for conical nozzles with cone angles, θ, varying from 0° (straight tube) to 30°. The (0,0) position denotes the upper edge of the nozzle. *J*_tot_ is the total molecule flow leaving the nozzle exit and the color code indicates the molecule fraction *J*/*J*_tot_. The cone geometries are shown to illustrate which areas are covered and hence inaccessible by the electron beam.

It is most useful to know how the nozzle geometry and the arrangement of the capillary with respect to the substrate influences the precursor flux at the substrate. The dependence of precursor flow on nozzle geometry (straight nozzle, bevelled nozzle with FEB access hole, closed capillary with FEB access holes), capillary angle, and shadowing by 3D objects have been studied by Friedli et al. [31,33]. More recently, it was shown that certain FEBIP scan strategies also lead to shadowing effects which can cause disruptions in surface flatness [23]. A specific simulation of a gas-flow distribution on a cantilever-based mass sensor enabled the estimation of the residence time of Me_3_PtCpMe on SiO_2_ [47]. With the released code the reader can include new nozzle geometries or substrate morphologies, for example a conical nozzle geometry as shown in Figure 7. With respect to the straight tube geometry the conical geometries are less practical for FEBIP in the molecular flow regime: Although cones “focus” the exiting molecules, they laterally reduce the maximum impinging flux area, physically cover the maximum flux area, and reduce the maximum impinging flux value slightly as shown by the *J*/*J*_tot_ contours. This behavior can be understood from the cosine-law redistribution of molecule collisions with the inner tube wall dominating the exiting precursor distribution in the molecular flow regime in contrast to the viscous flow experienced daily in water taps. Nonetheless, keeping the inner tube geometry straight and making the outer geometry conical could solve some space restrictions in the scanning electron microscope or allow to bring the nozzle closer to the substrate to increase the molecule impingement rate.

### Single gas species FEBIP model: etching or deposition

In the following sections, we outline continuum FEBIP models that can be utilized to simulate a wide range of experiments reported in the FEBIP literature. We start with the simple case of a precursor gas comprised of a single molecular species “a” (i.e., an etch or deposition precursor) that physisorbs to the substrate surface. In this case, the continuum FEBIP model is based on an expression for the rate of change of the adsorbate concentration *N*_a_ at each point *r* on the substrate surface. The expression for ∂*N*_a_/∂*t* is obtained by extending Equation 5 to account for the dissociation of adsorbates by electrons [16,19]:

[12]
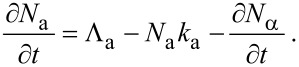


This equation is a sum of fluxes representing gas molecule arrival at the surface through adsorption (Λ_a_), adsorbate removal through thermal desorption (−*N*_a_*k*_a_), and adsorbate conversion to fragment species through electron induced dissociation (−∂*N*_α_/∂*t*). In Equation 12, *t* = 0 represents the time at which electron irradiation is activated in the model, and the initial adsorbate concentration 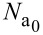 is given by Equation 7.

Most models of FEBIP assume that the adsorbate dissociation flux, ∂*N*_α_/∂*t*, is proportional to the product of the electron flux *f* and *N*_a_:

[13]
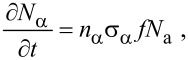


where *n*_α_ is the number of fragments α generated per dissociation event, and σ_α_ is the effective cross-section [3,18] for the generation of fragments that volatilize the substrate (in FEBIE) or deposit onto the substrate (in FEBID).

The steady state adsorbate concentration can be found by solving Equation 12 in the limit *t*→∞:

[14]
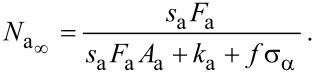


Substituting 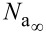 into Equation 13 gives the steady state growth rate 
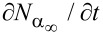
. The vertical growth rate ∂*h*/∂*t*, which is easily measured experimentally, is proportional to 
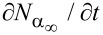
:

[15]
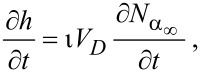


where *h* is the deposit height (or etch pit depth), ι is ±1 for deposition and etching, respectively, and *V*_D_ is the volume of a single molecule removed from or added to the substrate in the etch or deposition reaction.

The electron flux *f* can either be fixed at *f*_0_, or specified by an *r*-dependent function such as Equation 8 or Equation 9. Setting *f* to *f*_0_ is justified when simulating the growth rate or the deposit height (etch pit depth) at the beam axis (*r* = 0). We emphasize that the above equations are valid only in the reaction rate limited growth regime where net transport of adsorbates through diffusion is negligible. We also note that electron-stimulated desorption (ESD) [48–51] is assumed to be negligible. This assumption is typically justified because, for most adsorbates, the ESD cross-section σ_E_ lies in the range from 10^−7^ to 10^−2^ Å^2^ [50] (i.e., in general, σ_E_ *<<* σ_α_). However, if necessary, ESD can be incorporated in the above FEBIP model simply by adding the term (−σ_E_*fN*_a_) to Equation 12 [52].

**Figure 8:**
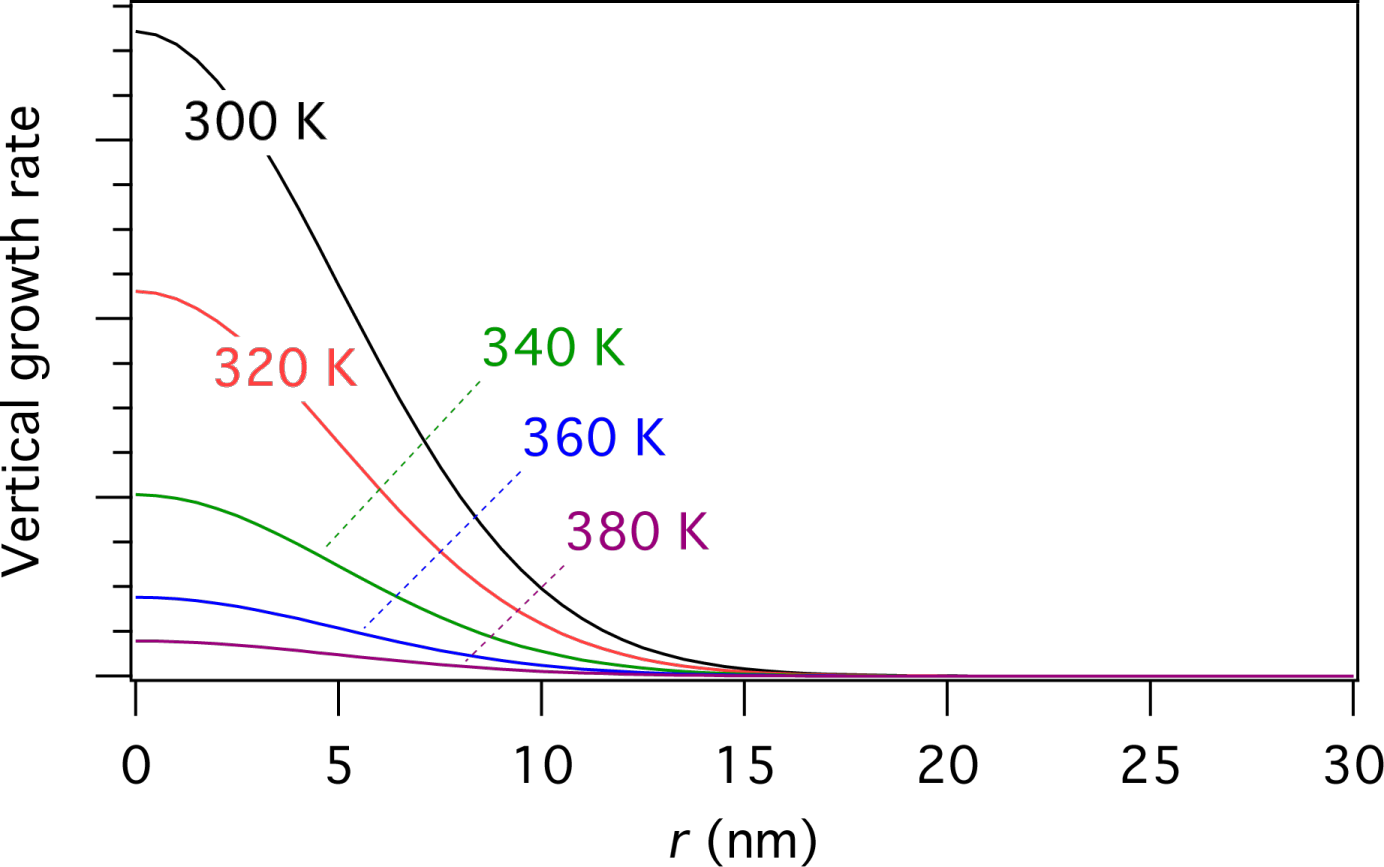
Steady state growth rate versus *r* calculated at a number of substrate temperatures using Equation 15 and a Gaussian electron flux profile (Equation 8, Ω = 5 nm).

Equation 15 can be used to calculate FEBIP growth rates as a function of experimental parameters such as the precursor gas pressure, electron flux and the substrate temperature. For example, Figure 8 shows a set of spatially resolved steady-state vertical growth rates calculated using a Gaussian electron flux profile and substrate temperatures of 300, 320, 340, 360 and 380 K. The growth rate decreases exponentially with temperature due to an increase in the thermal desorption rate *k*_a_ (given by Equation 4).

#### Dimensionless FEBIP models

We now introduce a number of dimensionless parameters, originally employed by Utke et al. [15], which are useful for describing adsorbate kinetics in FEBIP [1] and giving concise scaling laws for the lateral resolution of the FEBIP process.

*Irradiative depletion* 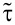 is a dimensionless parameter that quantifies the adsorbate concentration at the beam centre relative to the non-irradiated area. It is proportional to *f*_0_ and can be expressed as [53]:

[16]
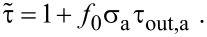


The *effective residence* time in the absence of electron irradiation (or outside the irradiated area) τ_out,a_ is given by:

[17]
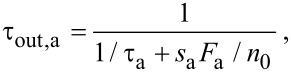


where τ_a_ is given by Equation 4, and *n*_0_ is the maximum possible adsorbate concentration (corresponding to one monolayer) in the absence of electron irradiation (i.e., *n*_0_ = 1/*A*_a_).

The steady-state concentration of adsorbates under an electron beam, given by Equation 14, and the steady-state vertical growth rate defined by Equation 15 can now be reformulated as:

[18]
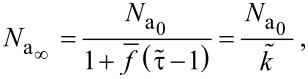


[19]
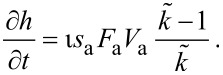


In the above, 
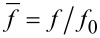
 is a dimensionless (normalized) version of the Gaussian or tophat electron electron flux profile given by Equation 8 or Equation 9, respectively, and 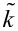 is given by:

[20]
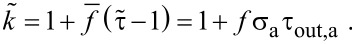


The lateral resolution can be quantified by the dimensionless parameter 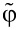, expressed as the ratio of the diameter of the deposit (FWHM_D_) and the electron beam (FWHM):

[21]
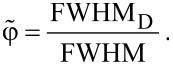


In the case of negligible surface diffusion, 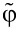 can be derived analytically yielding the 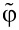-*vs*-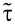 *scaling law of FEBIP resolution* for stationary Gaussian [15] and tophat electron beams:

[22]
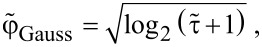


[23]
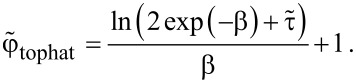


**Figure 9:**
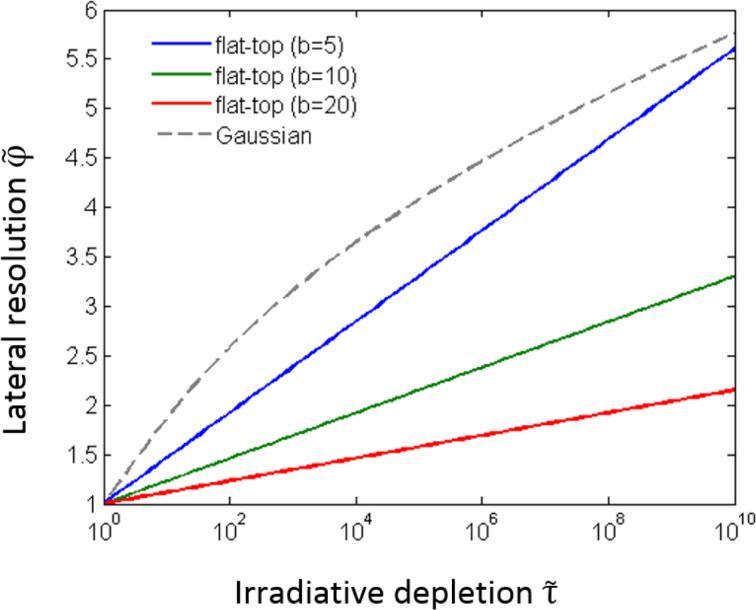
First FEBIP resolution scaling law for Gaussian and tophat electron beams.

The resolution scaling behavior defined by the above equations is shown in Figure 9. In the limit of very low irradiative depletion 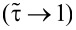 the resolution parameter is equal to 1, giving the highest possible FEBIP resolution (i.e., the smallest possible deposit size in the case of FEBID). In other words, the reaction limited (electron-limited) regime gives better lateral resolution than the mass transport limited (adsorbate-limited) regime. Deposits generated by tophat beams exhibit a less pronounced dependency of lateral resolution on irradiative depletion than those generated by Gaussian beams, and the dependence of 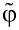 on 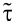 is a function of the blurring parameter β (see Equation 9). However, tophat profiles typically have a much larger beam diameter 2ξ compared to the FWHM of a Gaussian.

**Pulsed exposure:** To illustrate the use of dimensionless parameters in FEBIP models, we consider the case of FEBIP performed using a pulsed, Gaussian electron beam. Specifically, we consider the adsorbate kinetics during an electron pulse, under the assumption that that the surface is replenished entirely between consecutive electron pulses. When the electron beam is turned on, the adsorbate concentration *N*_a_ initially decays with time due to electron induced dissociation. The decay rate depends on the effective residence time τ_a,out_ and the irradiative depletion parameter 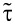. The resulting time-evolution of *N*_a_ is given by the solution to Equation 12, which can be expressed as:

[24]
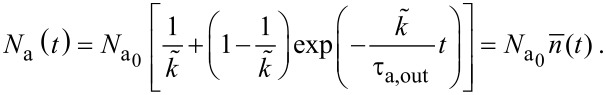


The corresponding vertical growth rate is proportional to *N*_a_(*t*) and the electron flux, and is given by:

[25]
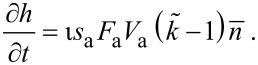


The lateral size of the growing structure or etch pit will evolve in time, yielding the 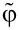-*vs*-*t law of pulsed FEBIP resolution*, expressed as the lateral resolution 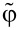, (neglecting surface diffusion) versus the electron beam exposure time *t* [1]:

[26]
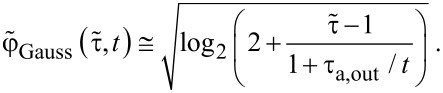


In the limit *t* → 0, 
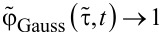
. In the steady state (*t* → ∞) this converges to the scaling law given by Equation 22.

We note that Equation 8 and Equation 9 for the beam profile do not account for lateral contributions of backscattered and forward scattered electrons as well as their generated secondary electrons to the final shape of the deposit or etch pit. The above model also does not account for the change of those contributions on the developing three-dimensional pillar and etch-pit geometry. These simplifications limit the applicability of the resolution scaling laws given by Equation 22, Equation 23, and Equation 26 as well as Equation 27 and Equation 28 to aspect ratios of roughly 1 to 4 for deposits and etch pits. A more detailed discussion on the applicability range can be found in [10]. For high-aspect ratio structures either a full Monte Carlo electron trajectory approach can be chosen [54] or the continuum equations outlined in our present work need to be solved on a curvilinear reference surface, see [13] and [55].

### Multiple gas species FEBIP model: Simultaneous etching and deposition

**Figure 10:**
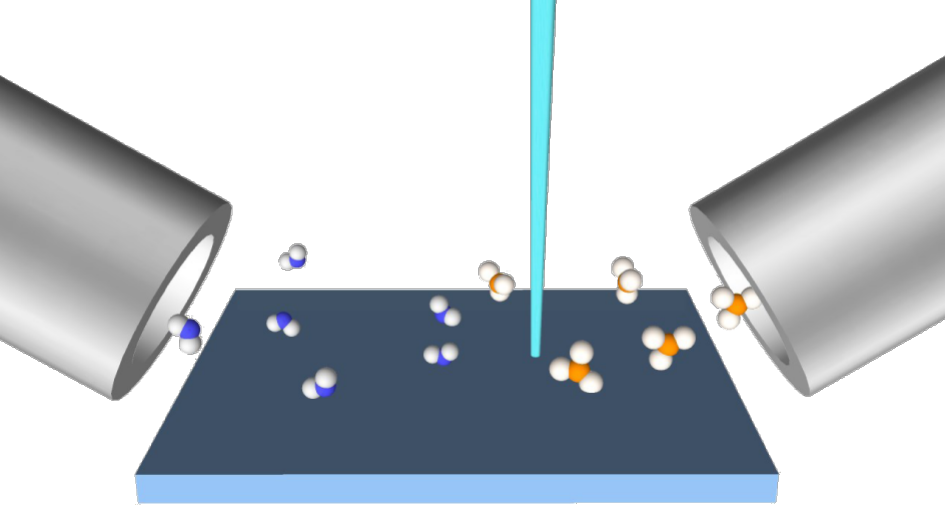
Illustration of two adsorbate FEBIP where the molecules are supplied by two capillaries and impinge with fluxes *F*_e_ and *F*_d_.

Deposition and etching arising from electron-induced dissociation of multiple adsorbed species is important in a number of experimental processes. Deliberate use of a deposition and etch precursor gas mixture, as shown in Figure 10, is often advantageous, such as in the deposition of high purity materials using a FEBID precursor and an oxygen-containing background gas [56–63], or a mixture of two FEBID precursors [4,8,64–66]. Unintentional deposition of carbonaceous films through electron-activated cross linking of hydrocarbon contaminants is a common problem in many etch processes [6,9,14].

Simultaneous FEBID and FEBIE can be simulated using models such as those described in [11], with adjustment to account for system-specific details such as the surface site competition behavior of the etch and deposit precursor molecules, adsorbate–adsorbate interactions, and whether the etch precursor can volatilize all or only some fraction of the deposited material. A specific example of this is H_2_O-mediated FEBIE of deposits formed from organometallic precursors, where only the deposited carbon (and not the metal) is etched.

Here, we illustrate how simultaneous etching and deposition can be incorporated into a continuum FEBIP model for the case of a mixture of H_2_O and C_10_H_22_ as the precursors for etching and deposition of carbon, respectively [11]. In this model, we assume that both etch and deposition precursor adsorbates (denoted by the subscripts “e” and “d”, respectively) physisorb to the substrate surface (Figure 2) in a competitive process, with the total surface coverage Θ being limited to 1 ML. The fluxes and coverages of the two species are given by the following set of Equation 29–Equation 30:

[29]
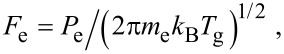


[31]
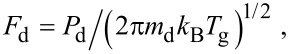


[32]
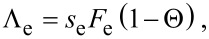


[33]
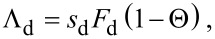


[30]
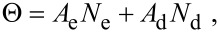


Here, Equation 29 and Equation 31 are directly analogous to Equation 1, Equation 32 and Equation 33 are analogous to Equation 2, and Equation 30 is equivalent to Equation 3, modified to account for the fact that the adsorbates “e” and “d” physisorb to the surface and compete for the same surface sites. Growth rates can be found by solving differential equations for the rate of change of the concentration of the species “e”, “d” and “D”, where the latter denotes molecules that are deposited as a result of FEBID:

[34]
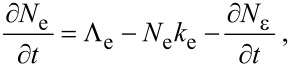


[35]
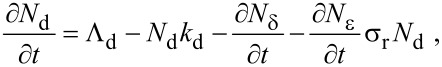


[36]
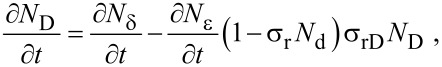


[37]
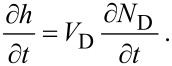


Equation 34 and Equation 35 are analogous to Equation 12, modified to account for the fact that FEBIE can volatilize the adsorbates “d” (i.e., the flux (∂*N*_ε_/∂*t*)σ_r_*N*_d_ represents FEBIE of the deposition precursor adsorbates). Equation 36 accounts for the simultaneous deposition of carbon through FEBID (∂*N*_δ_/∂*t*) and volatilization of the deposit via EBIE [(∂*N*_ε_/∂*t*)(1 − σ_r_*N*_d_)σ_rD_*N_D_* ], where (∂*N*_ε_/∂*t*)σ_r_*N*_d_ discounts those etch precursor fragments ε that are consumed in volatilizing the deposition precursor adsorbates “d”, and *N*_D_ must be capped at 1/*A*_d_ so that only molecules in the top monolayer of the deposit are available for volatilization by the fragments ε. The reaction cross-section σ_r_ (and σ_rD_) accounts for the effectiveness of collisions between the etch precursor fragments ε and the adsorbates “d” (or deposited molecules “D”) in contributing to etching [11]. Equation 37 is the vertical growth rate, and is directly analogous to Equation 15.

The fluxes ∂*N*_ε_/∂*t* and ∂*N*_δ_/∂*t* represent electron-induced dissociation of the adsorbates “e” and “d”, respectively, and are given by:

[38]
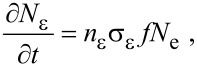


[39]
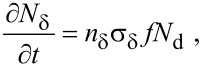


where the symbols have the same meanings as in Equation 13. The thermal desorption rates *k*_e_ and *k*_d_ have the same functional form as Equation 4:

[40]
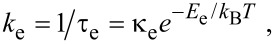


[41]
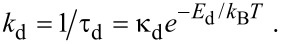


**Figure 11:**
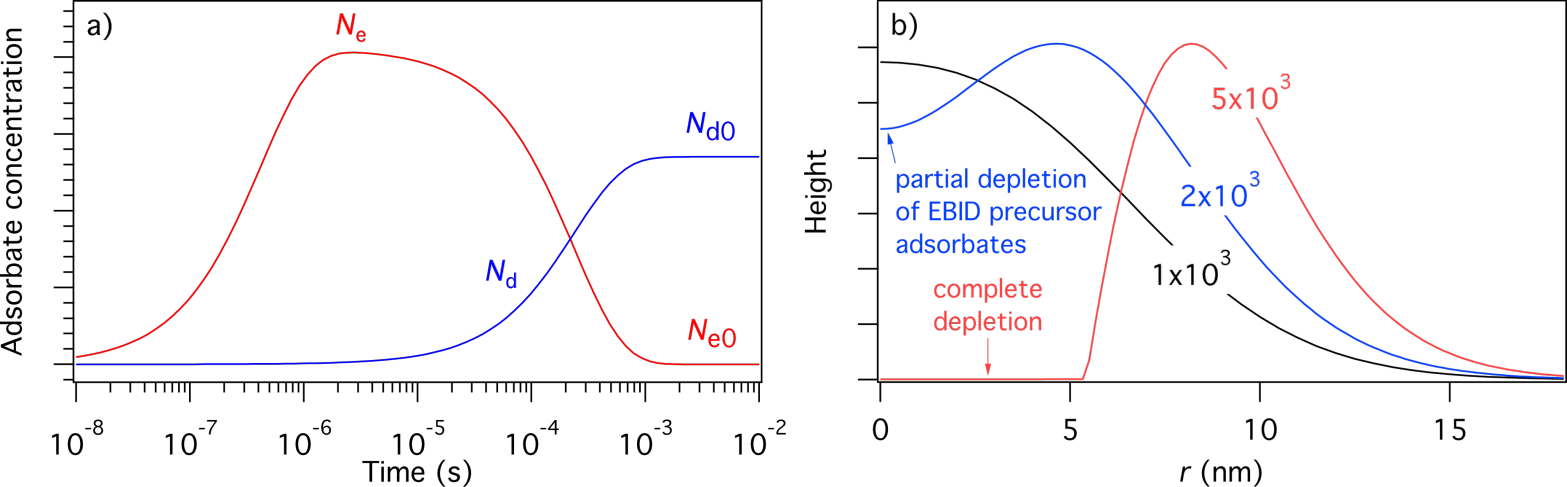
(a) Adsorbate concentrations *N*_e_ and *N*_d_ versus time, calculated in the absence of electron irradiation (here, *N*_e_ = *N*_d_ = 0 at *t* = 0). *N*_e0_ and *N*_d0_ are the steady-state adsorbate concentrations in the absence of electron irradiation, which are used as the initial adsorbate concentrations input into FEBIP models defined by Equation 29–Equation 39. (b) Deposit geometries simulated using a Gaussian electron beam with a standard deviation *(Ω)* of 5 nm and a maximum flux (*f*_0_) of 1 × 10^3^, 2 × 10^3^ and 5 × 10^3^ Å^−2^s^−1^. (Etch species = H_2_O, deposition species = C_10_H_22_, *P*_e_ = 1 Torr, *P*_d_ = 0.01 Torr, *s*_e_ = 1, *s*_d_ = 0.4, *E*_e_ = 0.56 eV, *E*_d_ = 1.0157 eV, κ_e_ = κ_d_ = 10^16^ s^−1^, σ_ε_ = σ_δ_ = 1 Å^2^, σ_r_ = σ_rD_ = *A*_d_ = 74 Å^2^, *T* = *T*_g_ = 300 K. All other model input parameters were the same as in [11].)

Initial concentrations of the species “e” and “d” (i.e., *N*_e0_ and *N*_d0_, respectively) can be found by solving Equation 34 and Equation 35 for the case of zero electron flux. The resulting time-evolution of surface coverage is more complex than that of the single gas species model shown in Figure 3, due to the nature of the surface site competition effect implemented in Equation 32–Equation 30. This is illustrated in Figure 11a for the case where *P*_e_ *>> P*_d_ and *E*_e_ *<< E*_d_. Initially, *N*_e_ increases much more rapidly than *N*_d_ because *P*_e_ *>> P*_d_ (and hence *F*_e_ *>> F*_d_). However, at times greater than ca. 10^−4^ s the increase in *N*_d_ causes a corresponding decrease in *N*_e_ because the two species compete for the same surface sites and *E*_d_ *>> E*_e_ (and hence τ_d_*<<* τ_e_).

The utility of the simultaneous FEBIE/FEBID model defined by Equation 34, Equation 35 and Equation 36 is illustrated in Figure 11b, which shows a number of deposits simulated using a Gaussian beam (given by Equation 8), with a maximum flux (*f*_0_) of 1 × 10^3^, 2 × 10^3^ and 5 × 10^3^ Å^−2^s^−1^. At the lowest electron flux (*f*_0_ = 1 × 10^3^ Å^−2^s^−1^), the deposit shape is approximately Gaussian because the extent of adsorbate depletion by the beam is low. At the intermediate flux (*f*_0_ = 2 × 10^3^ Å^−2^s^−1^), the central region of the deposit contains an indent caused by preferential depletion of the FEBID precursor adsorbates “d”. At the highest electron flux (*f*_0_ = 5 × 10^3^ Å^−2^s^−1^), FEBIE dominates in the central region of the deposit due to complete depletion of “d”. The preferential depletion of species “d” is a direct consequence of the slower replenishment rate caused by the fact that *P*_e _*>> P*_d_. The difference between the replenishment rates of “e” and “d” is illustrated in Figure 11a (at times shorter than ca. 10^−5^ s).

We emphasize that the changes in deposit geometry seen in Figure 11b are caused by a competition between simultaneous FEBIE and FEBID processes, and are not caused by adsorbate diffusion (which is ignored by Equation 29–Equation 39). This competition and the resulting phenomenon of electron flux controlled switching between etching and deposition have been discussed in detail in [11,14]. The additional effects of adsorbate diffusion can be investigated using a version of the above model that incorporates diffusion (discussed below).

### Multiple adsorption states: Thermally activated chemisorption

**Figure 12:**
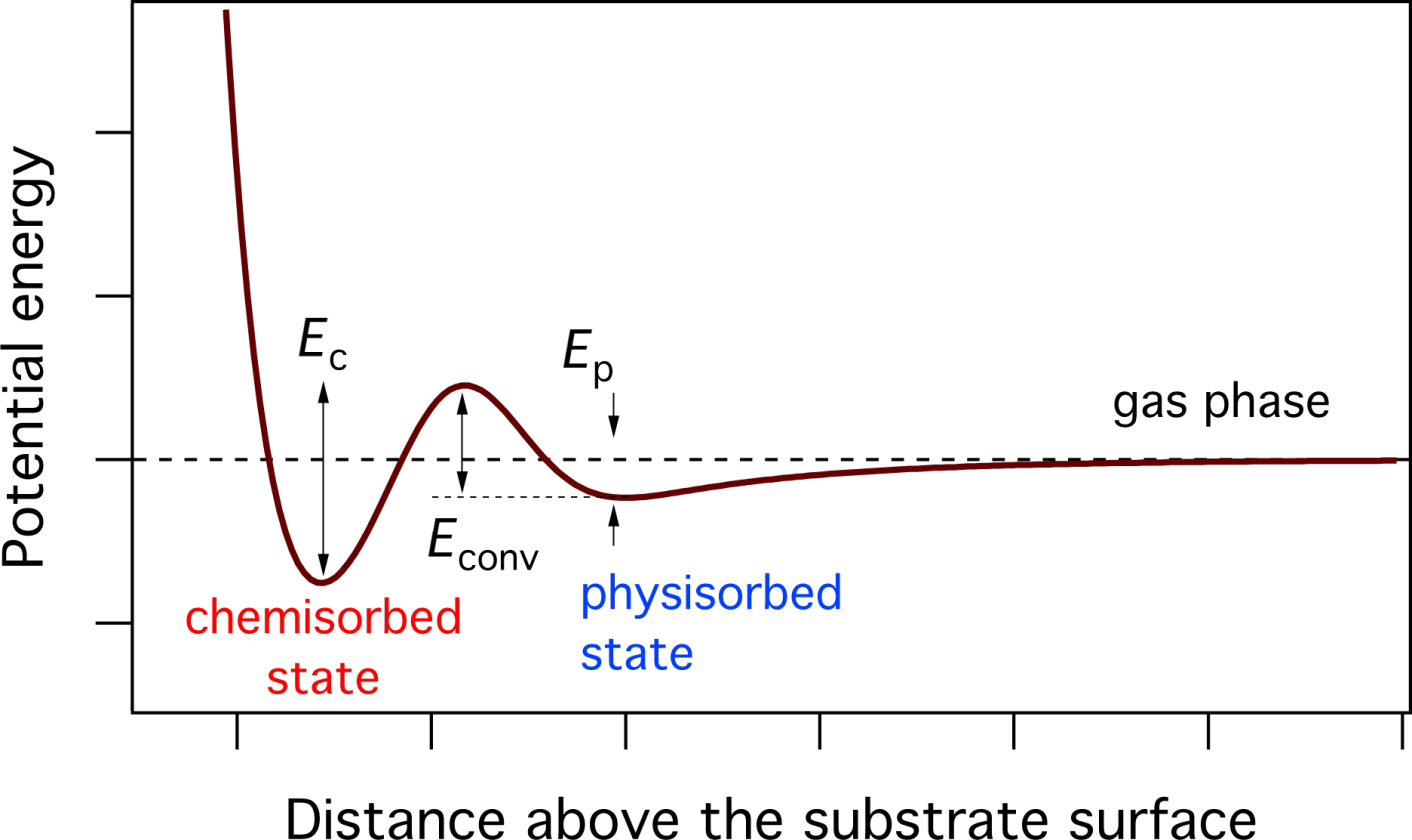
Potential energy diagram for the case of chemisorption governed by a potential well of depth *E*_c_ and an activation barrier of height *E*_conv_. Also shown is a physisorption potential well of depth *E*_p_. Modified from [5].

The FEBID growth rate typically decreases with increasing substrate temperature (as illustrated by Figure 8) due to thermal depopulation of the physisorbed state shown in Figure 2. However, recently it was shown that activated chemisorption can give rise to a more complex dependence of the growth rate on *T* due to thermal population and depopulation of chemisorbed states [5,67]. The simplest case of a system that can undergo physisorption and activated chemisorption can be described by the potential energy diagram shown in Figure 12 [68], comprised of a physisorbed state (denoted “p”) and a chemisorbed state (denoted “c”) separated by an activation barrier for conversion between these states. The respective physisorption and chemisorption potential wells have depths *E*_p_ and *E*_c_, and the activation barrier has height *E*_conv_.

Chemisorption is typically a dissociative process in which the activation barrier represents the energy needed to fragment the precursor molecule. For example, O_2_ chemisorbs onto numerous surfaces through the thermally activated reaction O_2_ → 2O, where O_2_ is in the gas phase or in the physisorbed state, and the two O atoms are in the chemisorbed state. Similarly, XeF_2_ can fluorinate many surfaces through decomposition pathways that generate chemisorbed F [17,69–71], and the FEBID precursor tetraethyl orthosilicate (TEOS, Si(OC_2_H_5_)4) chemisorbs onto SiO_2_ as a mixture of (SiO)_2_Si(OC_2_H_5_)_2_ and (SiO)Si(OC_2_H_5_)_3_ [72–74].

Chemisorption of gas phase precursor molecules can take place if their thermal energy is sufficiently high to surmount the barrier *E*_conv_ − *E*_p_ (see Figure 12):

[42]
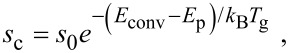


where *s*_c_ is the sticking coefficient for activated sticking of gas molecules into the chemisorbed state (in the limit of zero surface coverage), *s*_0_ is the preexponential factor (i.e., the sticking coefficient for the limiting case of *E*_conv_ − *E*_p_ = 0), and *T*_g_ is the temperature of the precursor gas. Similarly, molecules in the physisorbed state can surmount *E*_conv_ by gaining thermal energy from the substrate and populate the chemisorbed state at a rate *k*_conv_:

[43]
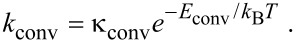


This pathway is more relevant to FEBID than chemisorption from the gas phase (i.e., Equation 42) because FEBID is typically implemented as a cold-wall deposition technique, whereby *T*_g_ is dominated by the temperature of the capillary used to deliver the precursor gas, and the substrate temperature *T* is used to control growth kinetics.

The thermal desorption rates of physisorbed (*k*_p_) and chemisorbed species (*k*_c_) are given by:

[44]
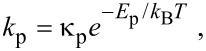


[45]
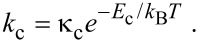


Activated chemisorption can be incorporated into the continuum FEBID model by accounting for the above transitions between the gas phase, physisorbed and chemisorbed states by replacing Equation 12 with a pair of equations for the rate of change of concentration of physisorbed molecules (∂*N*_p_/∂*t*) and chemisorbed molecules (∂*N*_c_/∂*t*) [5]:

[46]
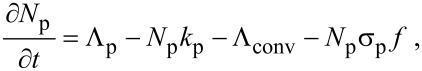


[47]
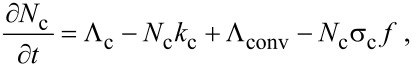


and by replacing Equation 13 with:

[48]
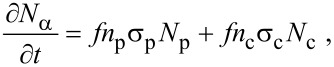


where *n*_p_ and *n*_c_ are the number of fragments generated per dissociation event of the physisorbed and chemisorbed molecules, respectively, assuming that both contribute to FEBID. Hence, the vertical growth rate is given by:

[49]
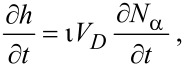


which assumes that the volumes of molecules deposited by the dissociation of physisorbed and chemisorbed adsorbates are both equal to *V*_D_.

The fluxes Λ_p_, Λ_c_ and Λ_conv_ correspond to physisorption from the gas phase, chemisorption from the gas phase, and transitions from the physisorbed to chemisorbed states, respectively:

[50]
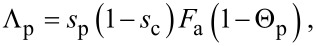


[51]
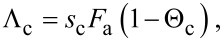


[52]
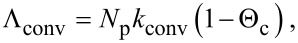


where *F*_a_ is the flux of precursor molecules “a”. The term (1 − *s*_c_) excludes gas molecules that are trapped in the physisorption potential well, and Θ_p_ and Θ_c_ are the coverages of physisorbed and chemisorbed adsorbates, each limited to 1 ML by:

[53]
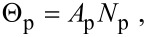


[54]
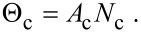


We note that energetic electrons can cause partial decomposition of precursor molecules and hence induce transitions from the physisorbed to the chemisorbed state. This effect is neglected here, but can be incorporated into FEBIP models as in [17] for the case of fluorination caused by decomposition of XeF_2_.

**Figure 13:**
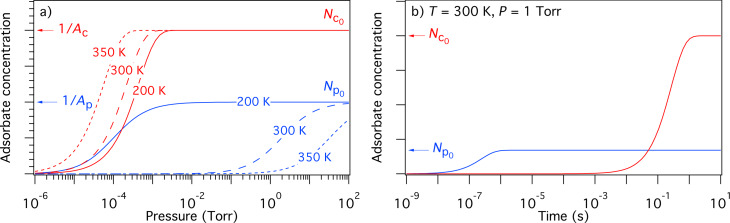
(a) Steady state concentrations of physisorbed 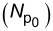 and chemisorbed 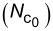 adsorbates versus pressure, calculated for the case of zero electron flux, and substrate temperatures of 200, 300 and 350 K. (b) Time-dependence of *N*_p_ and *N*_c_ calculated for a substrate temperature of 300 K and a precursor gas pressure of 1 Torr. (In all cases, gas temperature = 300 K; *E*_p_ = 510 meV, *E*_conv_ = 1.415 eV, *E*_c_ = 855 meV; *f* = 0; *A*_p_ = 2*A*_c_ and *n*_c_ = 2 to represent a dissociative chemisorption process in which the precursor molecule fragments into two chemisorbed molecules. All other model input parameters were the same as in [5].)

The initial concentration of physisorbed 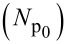 and chemisorbed 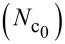 species is found by solving Equation 46 and Equation 47 for the case of zero electron flux. An example of the pressure dependence of 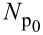 and 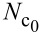 is shown in Figure 13a for a particular set of the energies *E*_p_, *E*_conv_ and *E*_c_ (corresponding to the chemisorption of TEOS onto SiO_2_ [5]), and substrate temperatures of 200, 300 and 350 K. The residence times of physisorbed and chemisorbed adsorbates are given by 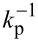 and 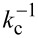, which are governed by the depths of the chemisorption and physisorption potential wells shown in Figure 12. At a substrate temperature of 350 K, chemisorbed states are rapidly populated through the physisorbed state (Equation 43), but depopulate slowly as *E*_conv_ *<< E*_c_ (i.e., *k*_conv_ *>> k*_c_). Conversely, physisorbed states are rapidly depopulated because *E*_p_ is smaller than both *E*_conv_ and *E*_c_. Consequently, 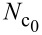 saturates at the monolayer limit of 1/*A*_c_ at a much lower pressure than 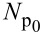 saturates at the monolayer limit 1/*A*_p_. At the lower substrate temperatures of 300 and 200 K, *k*_conv_, *k*_p_ and *k*_c_ are reduced through Equation 43, Equation 44 and Equation 45, yielding the adsorption isotherms shown in Figure 13a.

It is important to note that the rate of chemisorption is always limited by the activation barrier shown in Figure 12. Hence, the rate at which physisorbed states are populated (and replenished during FEBID) is always greater than the rate at which chemisorbed states are populated. This is illustrated by the plots of *N*_p_(*t*) and *N*_c_(*t*) shown in Figure 13b where, starting with a depopulated surface, the physisorbed species take ca. 10^−6^ s to populate the surface and reach a steady state, whereas it takes the chemisorbed species ca. 1 s to reach a steady state.

**Figure 14:**
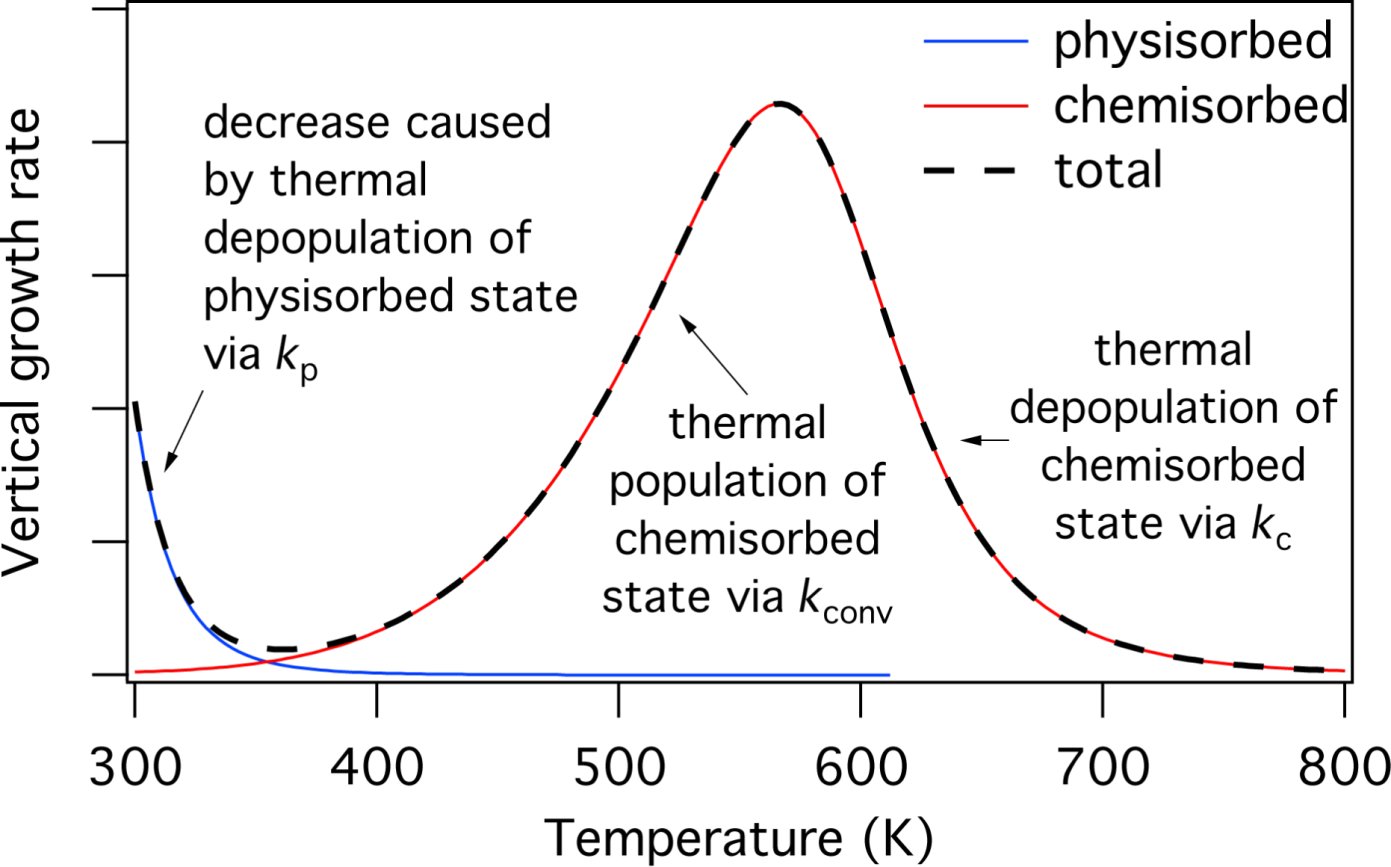
Steady-state vertical growth rate versus substrate temperature for a precursor that undergoes activated chemisorption described by the potential energy digram shown in Figure 12. The blue and red curves show contributions arising from electron induced dissociation of physisorbed and chemisorbed adsorbates, respectively. Modified from [5].

Equation 49 can be used to calculate the temperature-dependence of FEBID, as shown in Figure 14 for the case of TEOS [5]. The contribution to growth rate made by physisorbed adsorbates (i.e., *f*σ_p_*N*_p_ in Equation 48) decreases with increasing *T* due to an increase in the thermal desorption rate *k*_p_ (and hence a decrease in the adsorbate coverage *N*_p_). The chemisorption component (i.e., *f*σ_c_*N*_c_ in Equation 48) is negligible at room temperature because *E*_conv_ *>> k*_B_*T*_g_ and *E*_c_ − *E*_p_ *>> k*_B_*T*. It is characterized by a peak produced by thermal conversion of adsorbates from the physisorbed to the chemisorbed state (i.e., the low-temperature tail of the peak) and desorption from the chemisorbed state (i.e., the high-temperature tail), respectively. The general temperature dependence seen in Figure 14 (i.e., a decrease in the physisorption component followed by a peak caused by chemisorption) exists only if *k*_conv_ *<< k*_p_ *<< k*_c_ [5]. A practical benefit of performing FEBID at elevated temperatures (whereby, in this case, *T* ≥ 350 K) is that the deposits have much higher purity than those fabricated at room temperature [5].

### Role of multiple reaction products in electron beam induced etching

Etch processes such as XeF_2_-mediated FEBIE of SiO_2_ [12] and NF_3_ FEBIE of Si [2] proceed through multiple chemical pathways that involve a number of reaction products. Here, we use the case of NF_3_ FEBIE of Si [2] to illustrate how such processes can be incorporated in continuum FEBIE models.

FEBIE of Si using NF_3_ as the precursor gas involves the reaction products SiF, SiF_2_, SiF_3_, and SiF_4_, each of which can be dissociated by electrons, or can desorb from the substrate surface. For example, SiF_2_ can either gain F to form SiF_3_, dissociate to form SiF and F, or it can desorb (and hence give rise to etching by removing a Si atom):


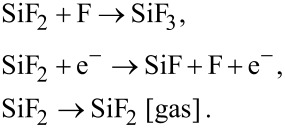


Multi-step FEBIE reactions can be modeled using a set of differential rate equations that account for each molecular species at the substrate surface, which in this case are the NF_3_ precursor adsorbates (denoted by “a”), F radicals *(α)*, and the reaction products SiF, SiF_2_, SiF_3_, and SiF_4_:

[55]
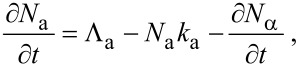


[56]
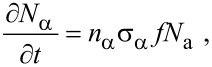


[57]
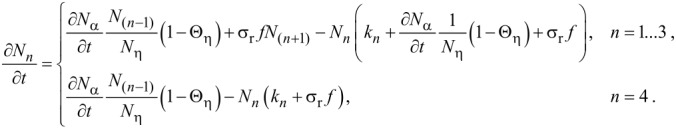


**Figure 15:**
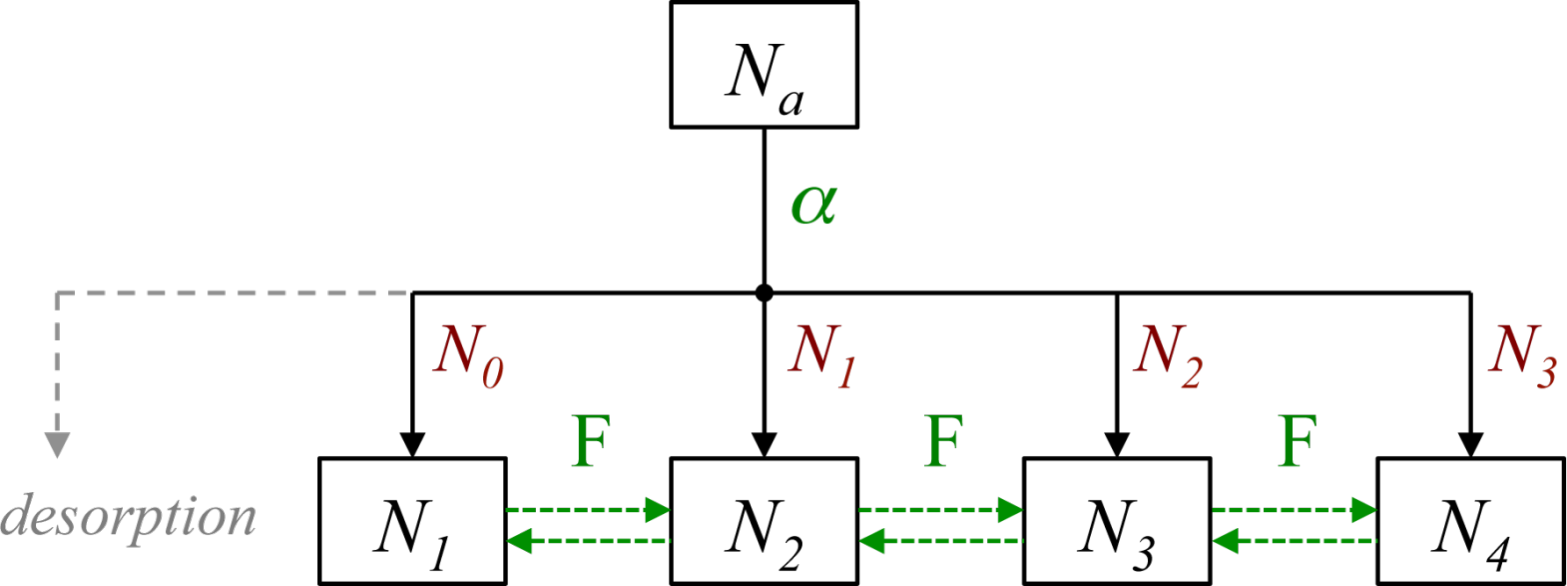
Flowchart showing how F radicals (represented by α) generated by electron induced dissociation of NF_3_ adsorbates (represented by the adsorbate concentration *N*_a_) are converted into SiF*_n_* (*N_n_*) by reacting with SiF*_n_*_−1_ (*N_n_*_−1_, shown in red). Horizontal green arrows indicate conversion of SiF*_n_* species by the addition and removal of F. The flowchart illustrates why *m* in Equation 59 ranges from 0 to 3 (corresponding to *N_n_*_−1_, shown in red), and why *n* in Equation 60 ranges from 1 to 4 (corresponding to *N_n_*, shown in black).

The symbols in Equation 55 and Equation 56 represent the same quantities as the corresponding symbols in Equation 12 and Equation 13 (see Table 1). In Equation 57, *N_n_* (*n* = 1–4) represents the concentration of SiF*_n_* molecules, σ_r_ is the cross-section for the electron-induced scission of the Si–F bond, and *k_n_* is the desorption rate of SiF*_n_*:

[58]
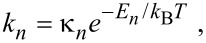


where *E_n_* is the binding energy of SiF*_n_*. *N*_η_ is the concentration of surface sites at which F can bond to a Si atom, and Θ_η_ is the coverage of surface sites occupied by F (see below in Figure 15):

[59]
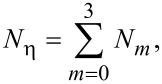


[60]
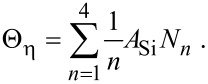


The term (1/*n*)*A*_Si_ limits the concentration of fluorinated Si atoms to one monolayer, *m* is an integer with lower and upper limits of 0 and 3 because an unfluorinated Si atom (designated by *m* = 0) can react with F to form SiF, whereas SiF_4_ (designated by *m* = 4) cannot gain an additional fluorine atom. The integer *n* is bound by 1 and 4 because the total coverage of sites occupied by F must account for SiF, SiF_2_, SiF_3_ and SiF_4_ species. *N*_0_ (the concentration of unfluorinated Si atoms at the surface) is given by:

[61]
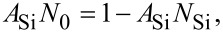


where *A*_Si_ is the area of a single Si surface site, and *N*_Si_ is the concentration of fluorinated Si sites:

[62]
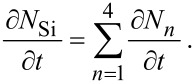


The vertical etch rate is governed by the desorption rate of SiF*_n_* molecules:

[63]
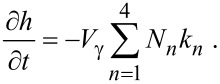


Models such as the above (Equation 55–Equation 63) are typically based on a number of simplifying assumptions in addition to those made in simple FEBID models. This particular model assumes that all available F radicals generated by electron-induced dissociation of NF_3_ are converted to SiF*_n_* by reacting with SiF*_n_*_−1_, and that any surplus fluorine atoms desorb as shown in Figure 15 (the desorption is assumed instant, hence the absence of a desorption flux in Equation 56). The probability of a reaction between F and each species SiF*_n_*_−1_ is assumed equal and the total F coverage is limited to 1 ML, hence the term (*N_n_*_−1_/*N*_η_)(1 − Θ_η_) in Equation 57 that governs the partitioning of the available F radicals illustrated by the flow chart shown in Figure 15. It is also assumed that σ_r_ is independent of *n*. Hence, the flux σ_r_*fN_n_*_+1_ represents the creation of SiF*_n_* through electron-induced dissociation of SiF*_n_*_+1_, and −*N_n_*σ_r_*f* the consumption of SiF*_n_* through electron-induced dissociation of SiF*_n_*. Finally, it is assumed that electrons are not consumed in any of the reactions, and that electron-stimulated desorption of all species is negligible.

**Figure 16:**
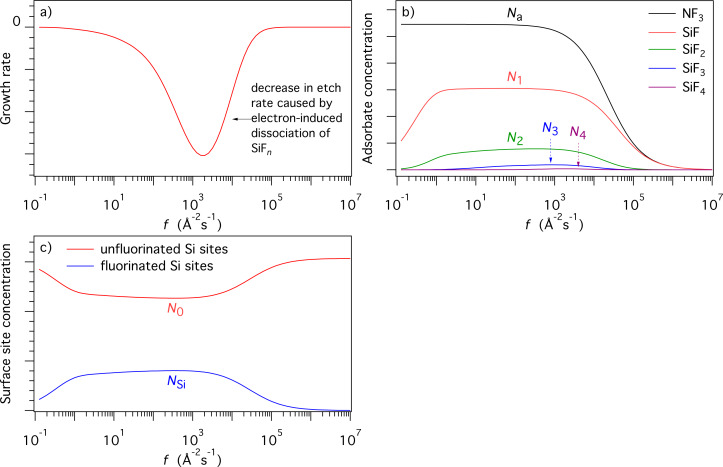
(a) Etch rate of Si calculated using Equation 63 as a function of electron flux *f*. (b,c) Corresponding steady state concentrations of NF_3_, SiF, SiF_2_, SiF_3_ and SiF_4_ molecules (*N_a_* and *N_n_*), fluorinated (*N*_Si_) Si sites, and unfluorinated (*N*_0_) and Si sites. (*T* = 100 K, *T*_g_ = 300 K. All other model input parameters were the same as in [2].)

The most significant consequence of the model defined by Equation 55–Equation 63 is that etching is inhibited at high electron fluxes, as seen in Figure 16a, due to electron-induced dissociation of SiF*_n_*. Specifically, this effect dominates when the dissociation rate of species *n* is much greater greater than the corresponding thermal desorption rate (i.e., σ_r_*f >> k_n_*). The simulations presented here were performed using model input parameters appropriate for cryogenic NF_3_-mediated FEBIP of Si performed at a substrate temperature of 100 K [2]. The model can be used to calculate a number of quantities such as the concentrations of NF_3_, SiF, SiF_2_, SiF_3_ and SiF_4_ molecules shown in Figure 16b, and the concentrations of unfluorinated (*N*_0_) and fluorinated (*N*_Si_) Si sites shown in Figure 16c.

### Special cases

The models outlined above can be used (either directly or in modified form) to simulate most processes reported in the FEBIP literature. Here, we summarize a few special cases for which the models require modification. We include these for completeness, and refer the reader to the cited papers for detailed descriptions of the changes that must be made to the above FEBIP models.

**Adsorbate depletion in high aspect ratio pits** [7]: The replenishment rate of precursor molecules consumed in FEBIE can be limited by the gas flow conductance of a growing etch pit. The replenishment rate affects the adsorbate concentration *N* which, in turn, affects the etch rate through equations such as Equation 15. Etch-pit conductance can be the most significant process limiting the FEBIE rate when fabricating high aspect ratio pits. As an etch pit grows during FEBIE, the conductance decreases, causing the etch rate to decrease with time as discussed in detail in [7].

**Dynamic surface site activation during FEBIE** [3]: Surface site activation caused by electron beam restructuring of the substrate (i.e., beam damage) can give rise to FEBIE of materials that can not be etched in their unmodified state. It can also alter etch kinetics, and cause the etch rate to increase with electron beam irradiation time.

**Electron beam induced surface functionalization and spontaneous decomposition of precursor molecules at the substrate surface**: These two processes have been modeled for the case of XeF_2_ [17] which can fragment through a dissociative chemisorption pathway, leading to fluorination of many surfaces [69–71,75]. The model in [17] is a variant of the above model of thermally activated chemisorption defined by Equation 42–Equation 54.

**Electron beam dwell time as a control parameter of the composition of materials deposited using a mixture of two precursor gases** [8]: This situation, depicted in Figure 10, can be modeled by solving rate equations for two FEBID precursor adsorbates, denoted by “A” and “B”, that compete for adsorption sites on the surface. The adsorbates are dissociated by electrons, producing non-volatile reactions products D_A_ and D_B_ (i.e., the deposit), and volatile reaction products V_A_ and V_B_:


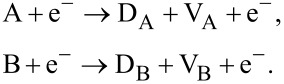


The evolution of the surface densities *n*_A_ and *n*_B_ of the two adsorbates is described by the following set of inhomogeneous first-order differential equations [8]:

[64]
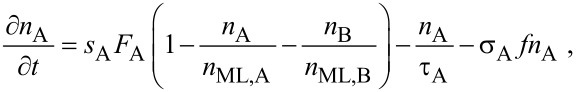


[65]
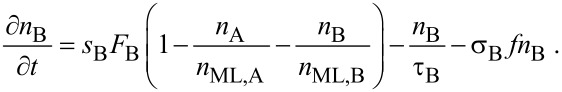


The generic solution of this set as a function of electron-beam exposure (dwell time) is given by:

[66]
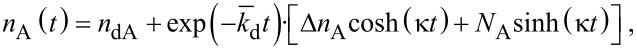


[67]
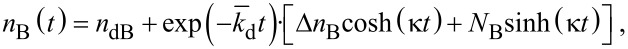


with the constants *n*_dA,B_, *k*_d_, Δ*n*_A,B_, κ defined in [8]. The dissociation yields *Y*_A,B_ are then obtained by integrating over the electron beam dwell time:

[68]
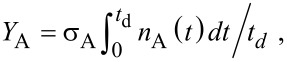


[69]
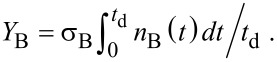


**Figure 17:**
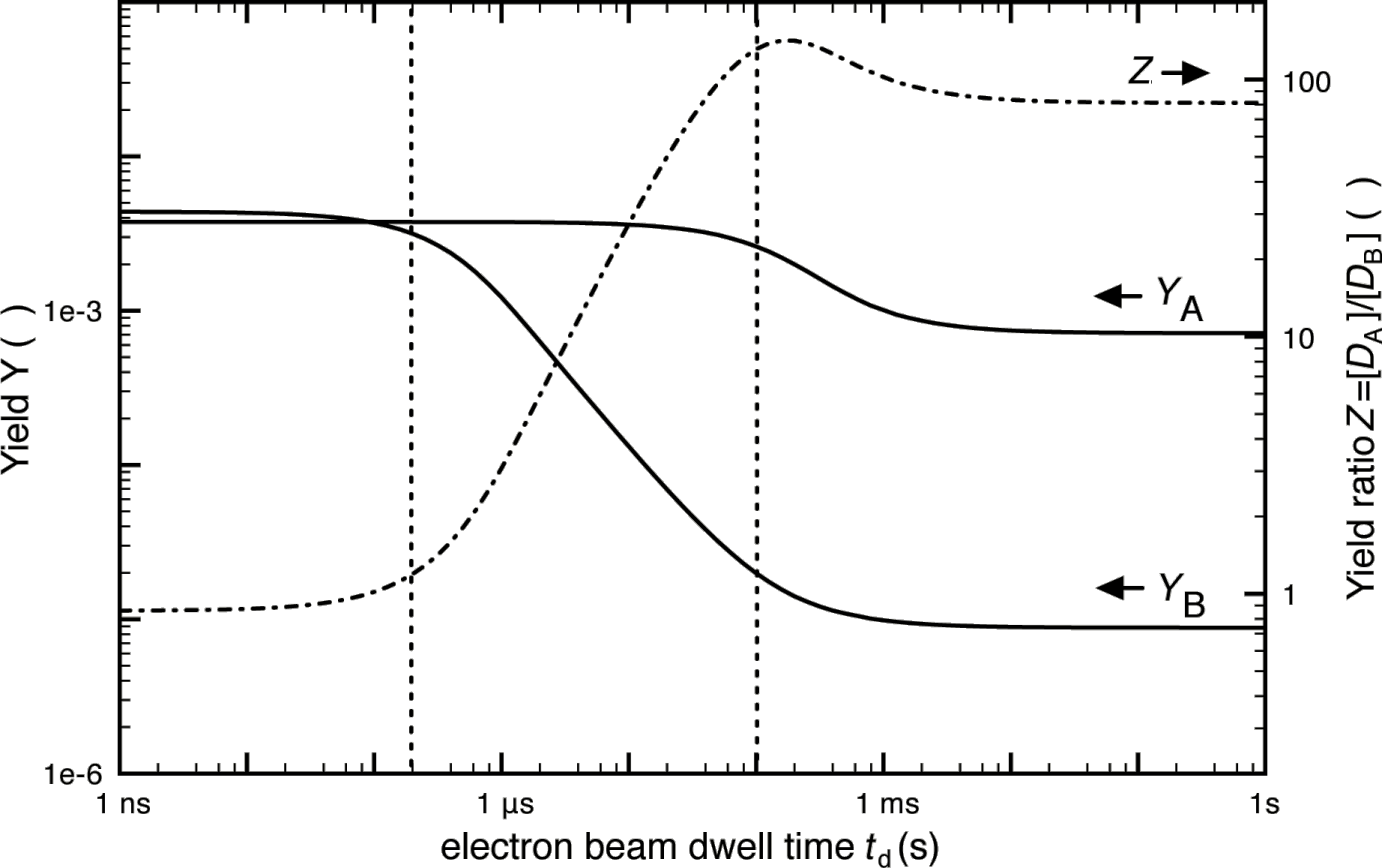
Calculated changes in dissociation yields *Y*_A_ and *Y*_B_ (per primary electron as defined in Equation 68 and Equation 69) and yield ratio *Z* versus electron beam dwell time. The example shown here was calculated for Co_2_(CO)_8_ and octanol as the deposition species “A” and “B”, respectively. The yield ratio *Z* is a measure for the ratio of non-volatile fragments of molecule “a” and “b” incorporated into the FEBID material. Taken from [78].

A graphical representation of Equation 68 and Equation 69 is shown in Figure 17. As the molecule fluxes, residence times, and dissociation cross-sections of molecules “A” and “B” are very likely different from each other, it can be seen that the composition (given by the magnitude *Z* on the right hand axis) of the deposits can be tuned by changing the electron beam dwell time per pixel. Equation 66 and Equation 67 hold for micrometer-sized deposits. For small-scale deposits surface diffusion can become important and different yields and compositions are obtained as function of exposure time, see Gabureac et al. [76–77]. Including surface diffusion into the multi-adsorbate model is straightforward, however, is at the expense of having a closed analytical solution.

### Diffusion

All of the above models are, strictly speaking, valid only in the reaction rate limited growth regime. They can be used to identify conditions under which depletion becomes significant, causing the system to transition into the mass transport limited regime (e.g., under intermediate and high electron flux conditions in Figure 11b, and the high electron flux portions of the curves in Figure 16). However, when the extent of depletion is significant (or, more precisely, when the net transport of adsorbates through diffusion is significant), the models must be modified to account for the diffusion of all mobile species at the surface. In the case of FEBID or FEBIE performed using a single species of physisorbed precursor molecules “a”, this is achieved simply by adding a diffusion term to Equation 12 [19]:

[70]
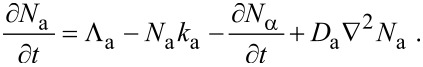


*D*_a_ is the diffusion coefficient, given by:

[71]
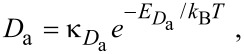


where 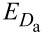 is the energy barrier for diffusion and 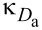 is the preexponential factor (i.e., the diffusivity in the limit 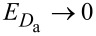).

The above approach for incorporating diffusion can also be applied to more complex FEBIP models, as we illustrate below. We note, however, that all of the continuum models discussed here assume a flat substrate surface. That is, the models track the time-evolution of deposits and etch pits made by FEBIP, but do not account for the effects of the resulting changes in surface geometry on adsorbate kinetics (e.g., adsorption to and diffusion along the evolving pillar sidewalls or etch pit sidewalls).

#### Gas mixtures

Equation 34–Equation 37 are used to model a gas mixture comprised of an etch precursor “e” and a deposition precursor “d” that simultaneously etch and deposit a material such as carbon. To account for the diffusion of the adsorbates “e” and “d” at the substrate surface, Equation 34 and Equation 35 are replaced by [11]:

[72]
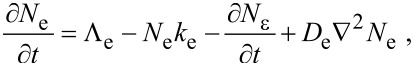


[73]
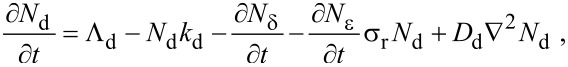


where *D*_e_ and *D*_d_ are the respective diffusion coefficients which have the same functional form as Equation 71:

[74]
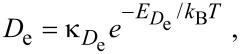


[75]
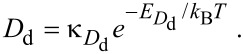


Equation 36 and Equation 37 remain unchanged since localized FEBIP requires the substrate temperature to be sufficiently low for diffusion of the deposited species “D” (e.g., carbon) to be negligible.

**Figure 18:**
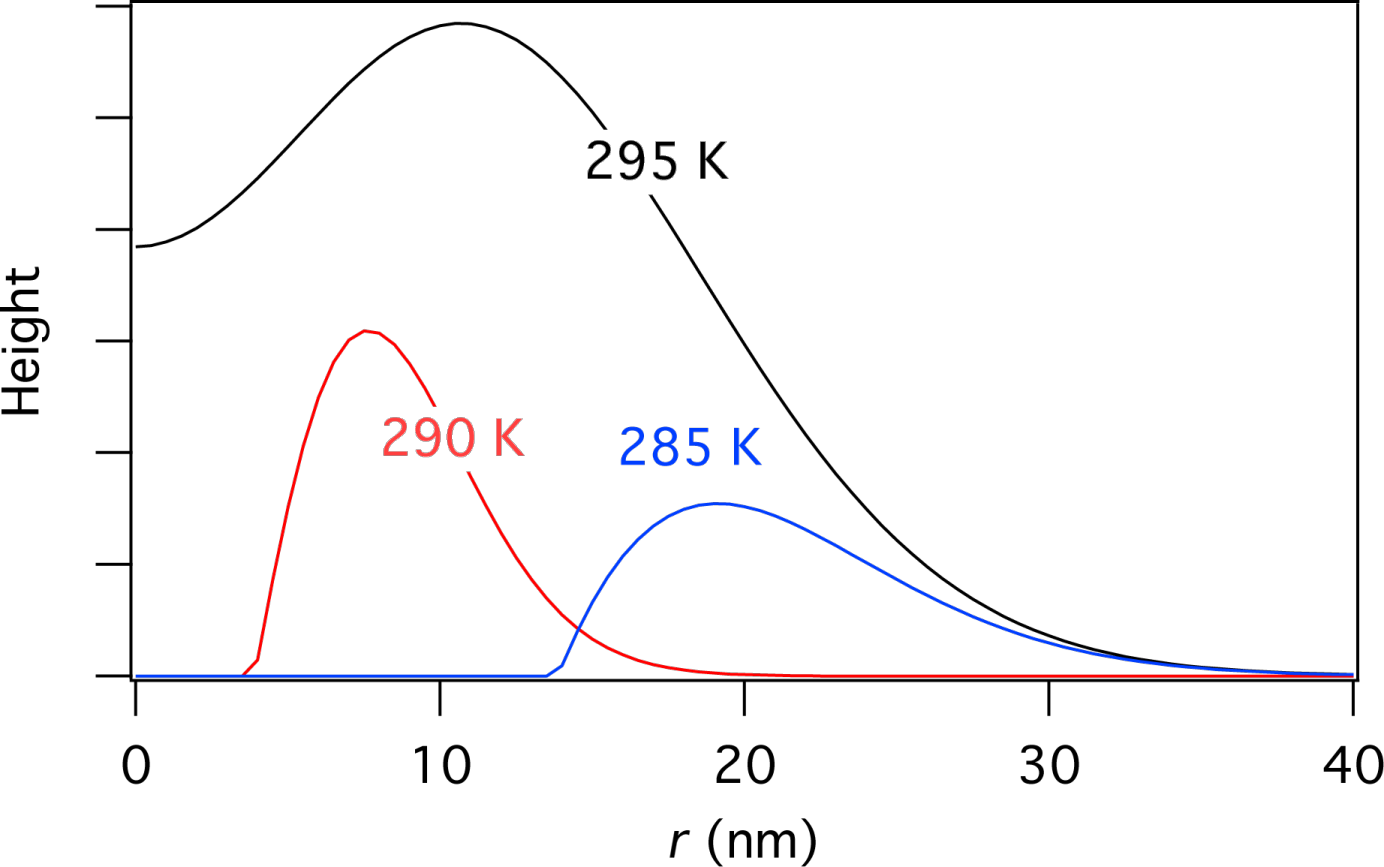
Deposit geometries analogous to those shown in Figure 11b, simulated using Equation 72 and Equation 73, a Gaussian electron-beam profile (Ω = 5 nm) and substrate temperatures of 285, 290 and 295 K. (Etch species “e” = H_2_O, deposition species “d” = C_10_H_22_, *f*_0_ = 6 × 10^4^ Å^−2^s^−1^, *P*_e_ = 1 Torr, *P*_d_ = 0.01 Torr, *E*_e_ = 0.56 eV, *E*_d_ = 1.0157 eV, σ_β_ = σ_γ_ = 1 Å^2^, *T*_g_ = 300 K. All other model input parameters were the same as in [11].)

Figure 18 shows deposit geometries simulated using Equation 72 and Equation 73, a Gaussian electron-beam profile and substrate temperatures of 285, 290 and 295 K. The dip in the center of each deposit is caused by the fact that etching dominates near the beam axis where the deposition precursor is preferentially depleted, while deposition dominates in the adjacent regions of low electron flux. The deposit geometry changes with temperature because an increase in *T* causes: (i) a decrease in *N*_e_ and *N*_d_ through thermal desorption (Equation 40 and Equation 41), and (ii) an increase in *N*_e_ and *N*_d_ through diffusion (Equation 74 and Equation 75). The dramatic change in geometry with increasing *T* shown in Figure 18 is largely due to reduced depletion of adsorbates “d” near the beam axis due to an increase in the rate of diffusion (Equation 75).

#### Thermally activated chemisorption

The model of activated chemisorption described by the potential energy diagram shown in Figure 12 can be extended to account for diffusion by adding diffusion terms to Equation 46 and Equation 47:

[76]
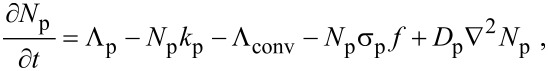


[77]
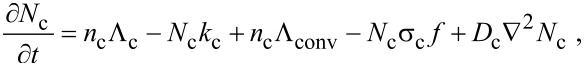


[78]
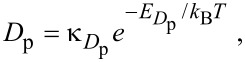


[79]
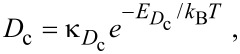


where *D*_p_ and *D*_c_ are the diffusion coefficients for physisorbed and chemisorbed adsorbates, respectively.

**Figure 19:**
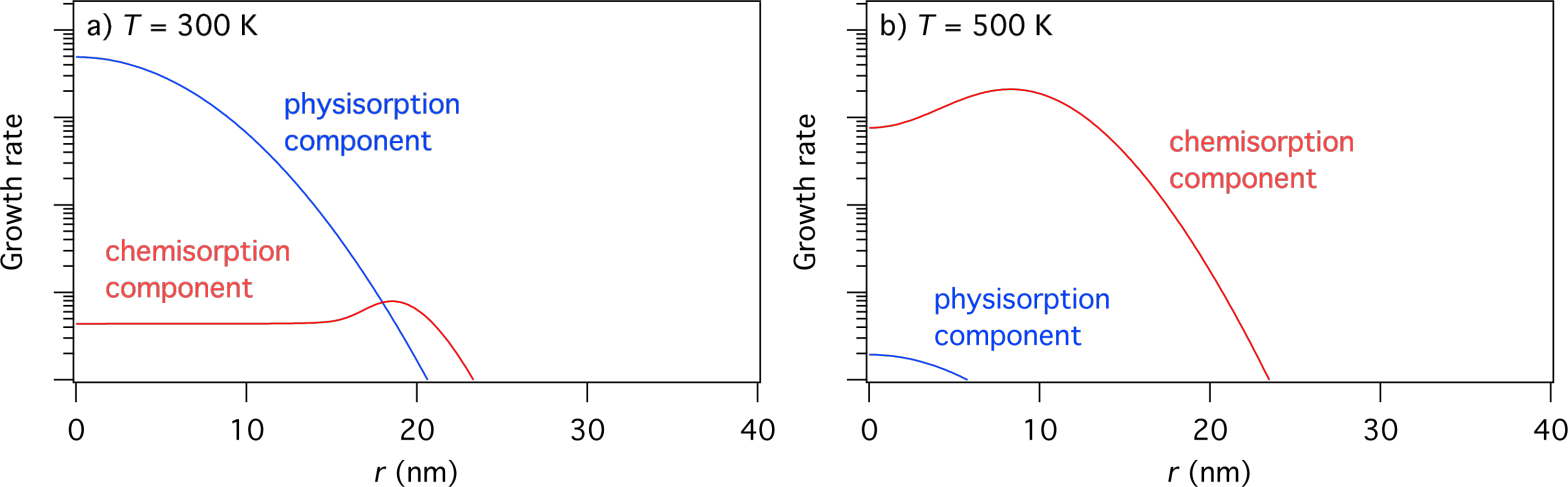
FEBID growth rates simulated using Equation 77 and Equation 78, a Gaussian electron-beam profile (Ω = 5 nm) and substrate temperatures of (a) 300 K and (b) 500 K. (Deposition precursor = TEOS, *f*_0_ = 10^3^ Å^−2^s^−1^, *P* = 0.1 Torr, *T*_g_ = 300 K, 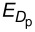 = 0.17 eV, 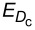 = 0.472 eV, 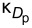 = 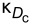 = 10^11^ Å^−2^s^−1^ all other model input parameters were the same as in [5].)

Figure 19 shows FEBID rates simulated using Equation 76 and Equation 77, a Gaussian electron-beam profile and substrate temperatures of 300 K and 500 K. At 300 and 500 K, FEBID is dominated by electron-induced dissociation of physisorbed and chemisorbed gas molecules, respectively (see Figure 14), because of preferential population of the two respective states shown in Figure 12.

#### Dimensionless FEBIP models

In this section, we incorporate surface diffusion into the dimensionless FEBIP model, introduced above through Equation 16–Equation 26.

The magnitude of adsorbate surface diffusion with respect to the FWHM size of the beam can be correlated by introducing the *dimensionless surface diffusion replenishment* parameter:

[80]
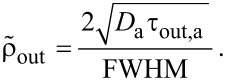


**Figure 20:**
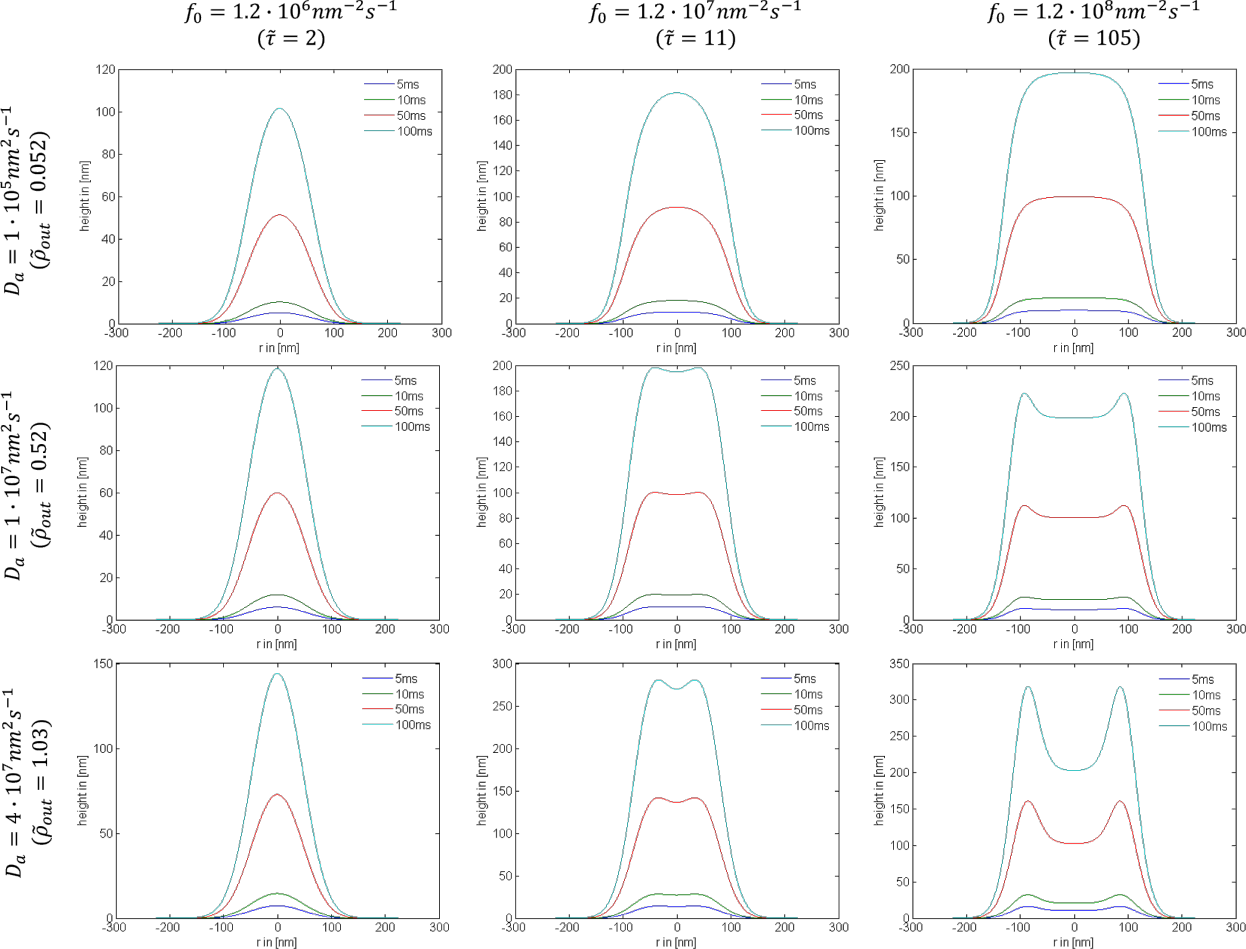
Examples of deposit geometries simulated using exposure times (*t*) of 5 ms; 10 ms; 50 ms; 100 ms, a Gaussian beam profile, and selected values of the maximum electron flux *f*_0_ and surface diffusion coefficient *D*_a_ (*s_a_* = 1, *F*_a_ = 10^4^ nm^−2^s^−1^, *n*_0_ = 2 nm^−2^, τ_a_ = 100 μs, σ_a_ = 0.013 nm^2^, *V*_a_ = 0.2 nm^3^ and FWHM = 100 nm).

**Figure 21:**
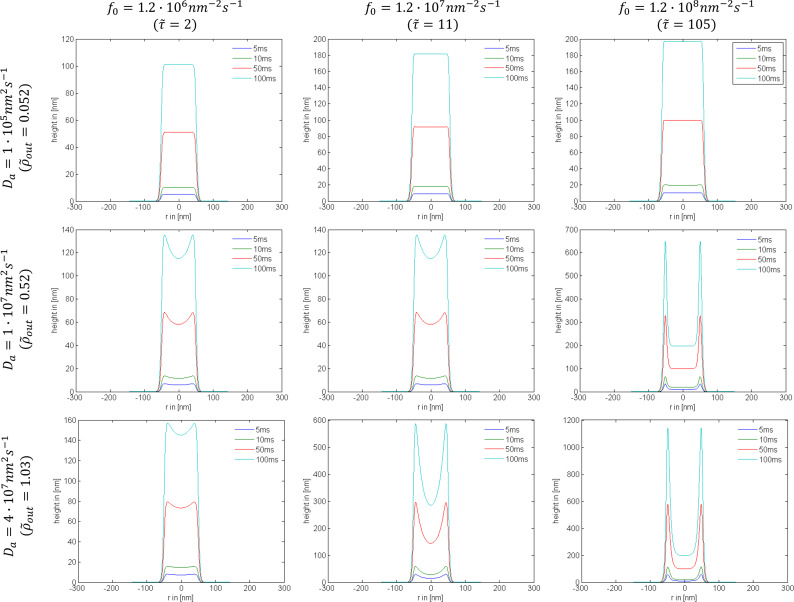
Examples of deposit geometries simulated using exposure times (*t*) of 5 ms; 10 ms; 50 ms; 100 ms, a tophat beam profile, and selected values of the maximum electron flux *f*_0_ and surface diffusion coefficient *D*_a_ (*s*_a_ = 1, *F*_a_ = 10 ^4^ nm^−2^s^−1^, *n*_0_ = 2 nm^−2^, τ*a* = 100μs, σ_a_ = 0.013 nm^2^, *V*_a_ = 0.2 nm^3^ and FWHM = 100 nm).

Substituting the irradiative depletion 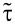 from Equation 16 and the surface diffusion replenishment 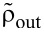 to the adsorbate rate equation, it becomes solely dependent on those two parameters and can be solved numerically [79]. Very small values of the surface diffusion replenishment parameter (e.g., 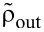 = 0.052) have a negligible effect on the lateral growth rate, as shown by row 1 of Figure 20 and Figure 21. However, large values (e.g., 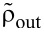 = 0.52) can alter the deposit geometries significantly, particularly in the case of a tophat electron beam (see rows 2 and 3 of Figure 20 and Figure 21). If the contribution of surface diffusion is very high (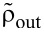 *>>* 0.1), adsorbate replenishment through this process dominates the growth kinetics. For example, when 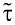 = 100, the deposit height is increased by a factor of 10 relative to the situation where adsorbate replenishment occurs only through adsorption from the gas phase.

The resolution parameter 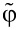 for a stationary Gaussian beam can be obtained from the deposit shape, using a numerical solution of Equation 70, and the expression for the growth rate given by Equation 15. The numerical result is very well approximated by the 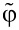-*vs*-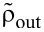 *scaling law of FEBIP resolution* formulated in [1]:

[27]
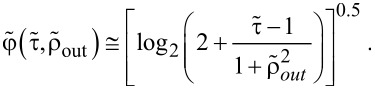


The above formula shows that even a relatively small surface diffusion contribution can lead to a decreasing in the deposit and etch pit size, and thus an improved lateral resolution. Once surface diffusion overcomes irradiative depletion, the deposit or etch pit diameter will approach that of the beam (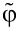 = 1), as discussed in [1].

**Pulsed exposure:** Equation 70 can be solved numerically, and the corresponding *general scaling law of FEBIP resolution*, including irradiative depletion, surface diffusion, and exposure dwell time, can be approximated by [1]:

[28]
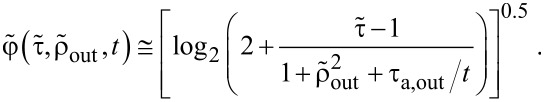


The dependencies of deposit geometry on the electron beam exposure time *t*, irradiative depletion 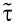 and surface diffusion path 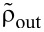 are shown in Figure 20 and Figure 21 for Gaussian and tophat beams, respectively. At short exposure times, depletion is negligible and the deposit shapes reflect the electron flux profiles. At long exposure times, the deposit geometry is determined by the extent of adsorbate depletion, and the relative contributions to adsorbate replenishment through diffusion and adsorption from the gas phase [1].

## Conclusion

In summary, we have reviewed continuum modeling techniques developed by the authors that can be used to simulate most processes reported in the FEBIP literature. Accompanying this article, we have released a software implementing most of these techniques at http://www.empa.ch/febipcode.

The software release consists of the following:

- An executable binary and C++ code of the GIS simulator for simulation of impinging molecule flux from capillary nozzles on plane substrates, which can be modified to generate Figure 7.
- A MATLAB notebook for single physisorbed gas species dimensionless FEBIP model that solve Equation 70 and Equation 49 for Gaussian electron beam (Equation 8). This was used to generate Figure 20.
- A MATLAB notebook for single physisorbed gas species dimensionless FEBIP model that solve Equation 70 and Equation 49 for tophat electron beam (Equation 9). This was used to generate Figure 20.
- Eight Mathematica notebooks, detailed below, that illustrate implementation of FEBIP models of varying complexity.

FEBIP models implemented in Mathematica notebooks:

1. Single physisorbed gas species initial coverage calculator, used to generate Figure 3.

2. Single physisorbed gas species FEBIP model based on Equation 12. This model was used to generate Figure 8.

3. Gas mixture FEBIP model based on Equation 34–Equation 41 and used to generate Figure 11a. For this simulation, initial coverages can be calculated by setting the electron flux to zero.

4. Gas mixture FEBIP model that incorporates diffusion, based on Equation 72–Equation 75 and used to generate Figure 18 and Figure 11b (in the latter, the diffusion coefficients were made negligible).

5. Thermally activated chemisorption initial coverage calculator used to generate Figure 13.

6. Thermally activated chemisorption, based on Equation 46–Equation 54 and used to generate Figure 14.

7. Thermally activated chemisorption with diffusion, based on Equation 76–Equation 79 and used to generate Figure 19.

8. EBIE with multiple reaction products, based on Equation 55–Equation 63 and used to generate Figure 16. Initial coverages can be calculated by setting the electron flux to zero.

Gaussian (Equation 8) or tophat (Equation 9) focused electron beams can be used by selecting the appropriate equations in each notebook.
